# Self-assembly as a key player for materials nanoarchitectonics

**DOI:** 10.1080/14686996.2018.1553108

**Published:** 2019-01-31

**Authors:** Katsuhiko Ariga, Michihiro Nishikawa, Taizo Mori, Jun Takeya, Lok Kumar Shrestha, Jonathan P. Hill

**Affiliations:** a WPI-MANA, National Institute for Materials Science (NIMS), Ibaraki, Japan; b Department of Advanced Materials Science, Graduate School of Frontier Sciences, The University of Tokyo, Kashiwa, Japan

**Keywords:** Nanoarchitectonics, self-assembly, interface, nanomaterial, 20 Organic and soft materials (colloids, liquid crystals, gel, polymers), 101 Self-assembly / Self-organized materials

## Abstract

The development of science and technology of advanced materials using nanoscale units can be conducted by a novel concept involving combination of nanotechnology methodology with various research disciplines, especially supramolecular chemistry. The novel concept is called ‘nanoarchitectonics’ where self-assembly processes are crucial in many cases involving a wide range of component materials. This review of self-assembly processes re-examines recent progress in materials nanoarchitectonics. It is composed of three main sections: (1) the first short section describes typical examples of self-assembly research to outline the matters discussed in this review; (2) the second section summarizes self-assemblies at interfaces from general viewpoints; and (3) the final section is focused on self-assembly processes at interfaces. The examples presented demonstrate the strikingly wide range of possibilities and future potential of self-assembly processes and their important contribution to materials nanoarchitectonics. The research examples described in this review cover variously structured objects including molecular machines, molecular receptors, molecular pliers, molecular rotors, nanoparticles, nanosheets, nanotubes, nanowires, nanoflakes, nanocubes, nanodisks, nanoring, block copolymers, hyperbranched polymers, supramolecular polymers, supramolecular gels, liquid crystals, Langmuir monolayers, Langmuir–Blodgett films, self-assembled monolayers, thin films, layer-by-layer structures, breath figure motif structures, two-dimensional molecular patterns, fullerene crystals, metal–organic frameworks, coordination polymers, coordination capsules, porous carbon spheres, mesoporous materials, polynuclear catalysts, DNA origamis, transmembrane channels, peptide conjugates, and vesicles, as well as functional materials for sensing, surface-enhanced Raman spectroscopy, photovoltaics, charge transport, excitation energy transfer, light-harvesting, photocatalysts, field effect transistors, logic gates, organic semiconductors, thin-film-based devices, drug delivery, cell culture, supramolecular differentiation, molecular recognition, molecular tuning, and hand-operating (hand-operated) nanotechnology.

## Graphical Abstract

**Figure:**
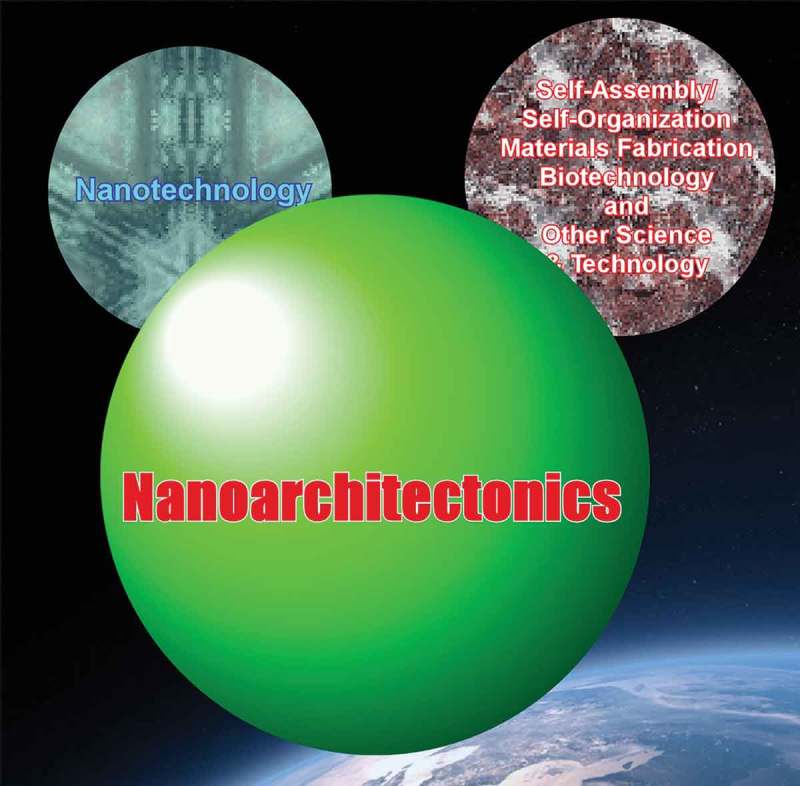

## 1. Introduction

Although cyber-space-related technologies such as information technology and artificial intelligence have made substantial recent progress, science and technology in physical space remain important for the development of human activities. By analogy with computational optimizations of efficiency and function of novel cyber technologies such as machine learning [1–5], the optimization of structure, combination, and organization of materials towards advanced functionality based on accumulated knowledge, theories, facts, experiences, and intuitions must continue [6–9]. Current societal demands including materials synthesis and production [10–14], energy storage and conversion [15–22], analyte sensing and detection [23–26], environmental remediation [27–30], and biological and biomedical applications [31–36] are currently being met through developments in the science and technology of advanced materials.

Further to historical efforts in organic synthesis and materials’ fabrication, functional optimization of materials involving structural regulation at the atomic/molecular scales and nanometre scales has been widely investigated and has been accompanied by the corresponding developments in observational techniques and instrumentation on those length scales [37–39]. While it is widely appreciated that nanotechnology plays a central role in materials developments involving nanoscale structures, nanotechnology also provides substantial benefits in the development of nanoscale observation and fabrication techniques including deepening our understanding of novel phenomena on the nanoscale and their guiding physical principles [40–43]. The construction of functional materials from nanoscale units requires essential contributions from non-nanotechnology fields such as supramolecular chemistry with self-assembly/self-organization [44–47], materials fabrications [48–50], and biotechnology [51–55]. Therefore, materials preparation from nanometric units in the nanoscale regime should to be conducted under a novel concept involving the agglomeration of nanotechnology concepts with the above-mentioned diverse research disciplines. The resulting concept is ‘nanoarchitectonics’ [56–58].

Emergence of the nanoarchitectonics concept can be seen as an inevitable event in the development of science and technology and its historical background can be traced in the scientific discourse [59]. The importance of the architectonics concept in the nanoscale sciences was first suggested in a 1999 article titled Architectonic Quantum Dot Solids by Heath and co-workers (University of California Los Angeles, UCLA) [60]. Subsequently, UCLA opened a dedicated research centre, Functional Engineered Nano Architectonics, in 2003. In the same year, Stefan Hecht of the Freie Universität Berlin, Germany, published a paper titled 'Welding, Organizing, and Planting Organic Molecules on Substrate Surfaces – Promising Approaches towards Nanoarchitectonics from the Bottom Up' [61], which is the first paper to mention explicitly the term nanoarchitectonics in the title of an academic paper published in an international scientific journal. Three years prior to those events in 2000, Masakazu Aono had organized the first International Symposium on Nanoarchitectonics Using Suprainteractions in Tsukuba, Japan [62]. This was possibly the first use of the term nanoarchitectonics in the scientific community. The initiator of the nanoarchitectonics concept, Masakazu Aono, in 2007 launched the World Premier International Research Center for Materials Nanoarchitectonics (WPI-MANA) at the National Institute for Materials Science in Tsukuba. In the same city, Toshimi Shimizu of the National Institute of Advanced Industrial Science and Technology also initiated a research centre, Interfacial Nanoarchitectonics. Thus, the concept of nanoarchitectonics was activated during a few years around the year 2000 in different countries all over the world.

**Figure 1.**
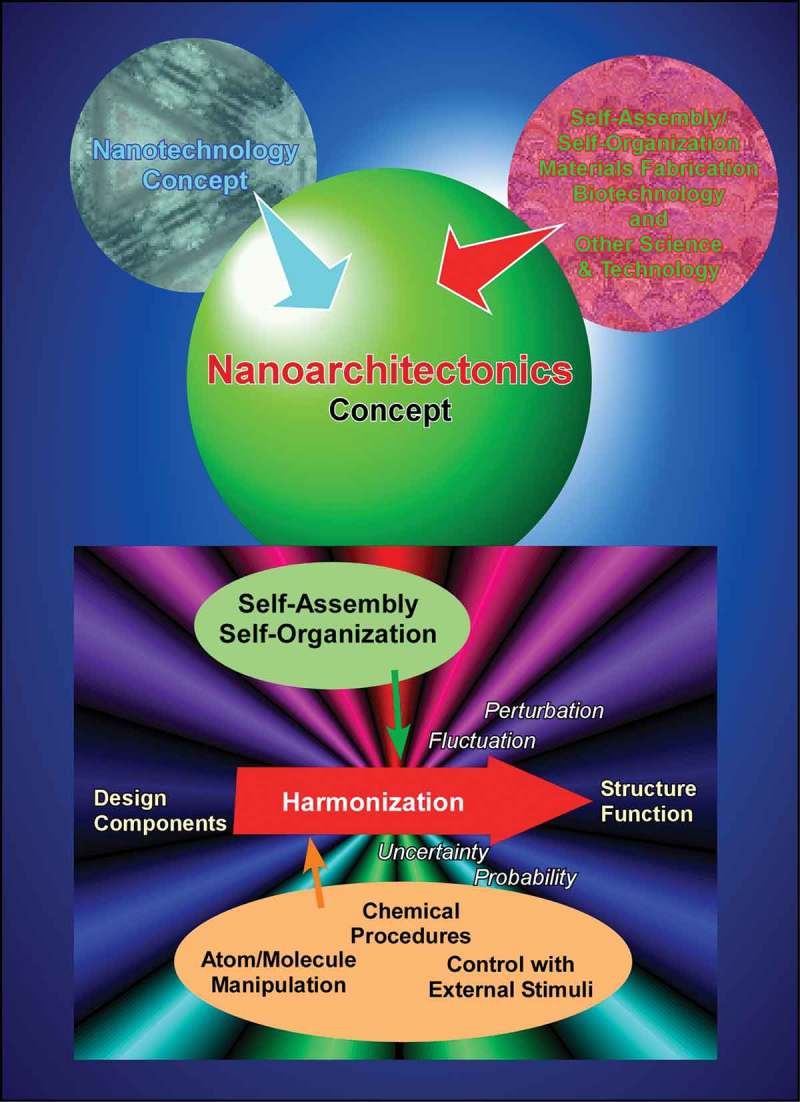
Outline of nanoarchitectonics concept: organization of nanoscale unit to functional materials and systems with some unavoidable uncertainties balanced harmonization of various factors.

According to Masakazu Aono, the guidelines of nanoarchitectonic strategies can be summarized as follows (Figure 1) [63]: (1) the organization of nanoscale unit structures results in creation of reliable materials and systems where some unavoidable uncertainties in nanoscale phenomena should be contained in balanced harmonization; (2) interactions between the nanometric components are often more important than the identities of the individual nano-components for the creation of novel functionalities; (3) unexpected functions might emerged through the assembly or organization of large numbers of nanoscale constituents. Nanoarchitectonic constructions can be undertaken using known strategies such as atom/molecule manipulation, chemical synthesis, chemical nano-manipulation, field-induced material control and self-assembly, and self-organization. However, in many cases, uncontrollable or unexpected interference originating from complex molecular interactions, thermodynamic perturbations, statistical uncertainties, and quantum effects cannot be avoided. Harmonization of these usually obstructive effects is one of the important keys [64]. Overall, the nanoarchitectonics concept represents a common standard strategy for materials and system creation in many research fields. Therefore, it has been used in a wide range of research fields and applications including materials production [65–68], materials fabrication [69,70], materials organization [71–74], supramolecular assemblies [75–78], device and physical systems [79,80], sensing [81–85], energy related applications [86–90], environmental strategies [91–94], and biological and biomedical applications [95–100].

Although nanoarchitectonics contains several different approaches for its construction mechanisms, self-assembling processes are often crucial involving a wide range of component materials. Therefore, an overview of emerging examples of self-assembling processes is meaningful in a re-examination of the recent progress in materials’ nanoarchitectonics [101–105]. Previously, in 2008, we published a comprehensive review titled 'Challenges and Breakthroughs in Recent Research on Self-Assembly' [106]. As a successor and supplement to that work, in the present review article, emerging examples of self-assembly research subsequent to 2008 (especially in a recent few years) are reviewed with additional aspects of the nanoarchitectonics concept. There is a large body of work based on self-assembly; hence, only selected research examples are introduced in this review. This review is composed of three sections: (1) an initial brief section where typical examples of self-assembly research are collected as an overview of matters discussed in this review; (2) the second section summarizes self-assemblies in various media from a general viewpoint; and (3) the last section focuses on self-assembly processes at interfaces. The examples presented demonstrate the strikingly wide possibilities and future potentials of self-assembly processes and their important contributions to materials nanoarchitectonics.

## 2. Typical research examples of self-assembly as an outline

**Figure 2.**
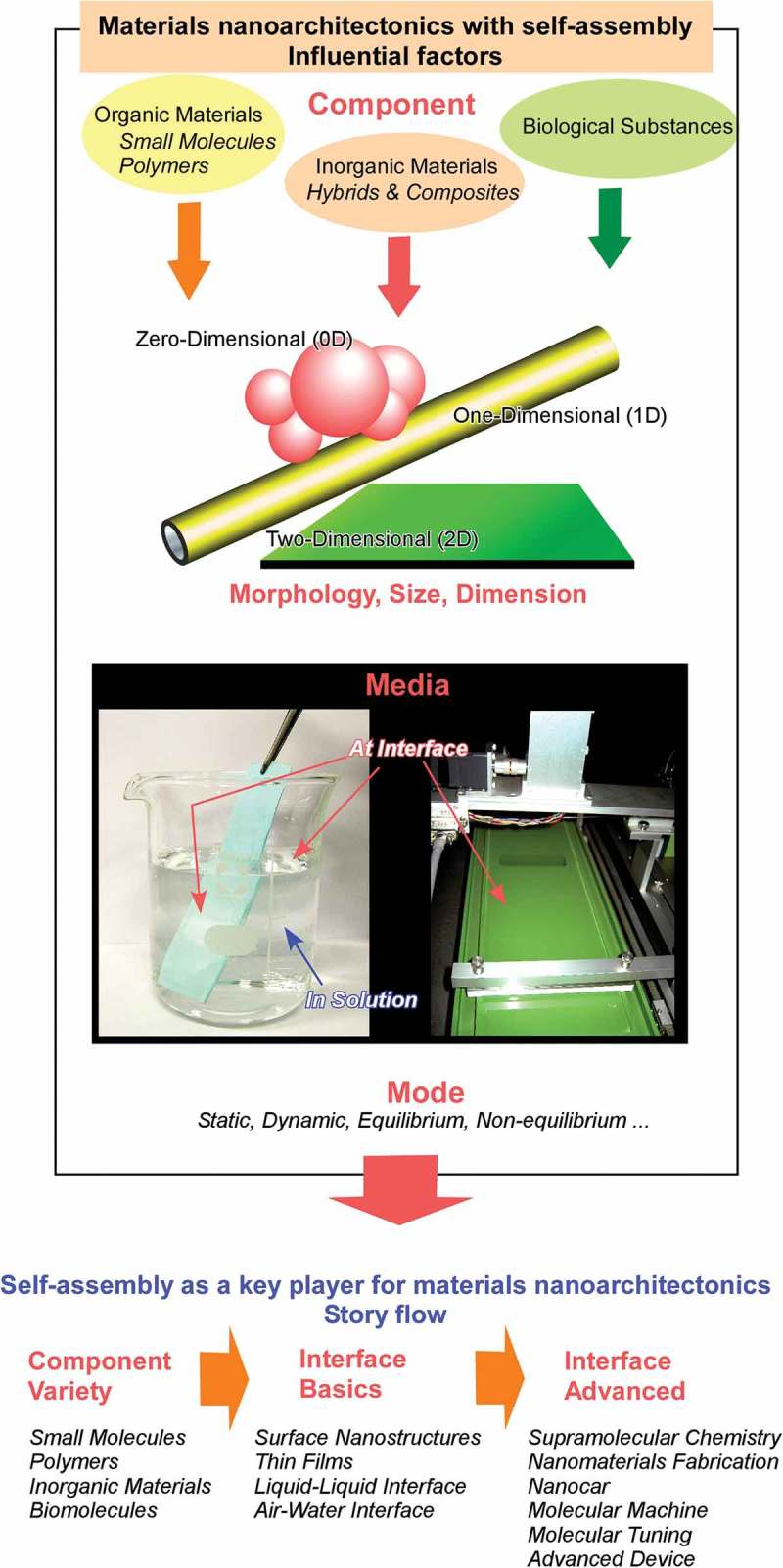
Outline of self-assemblies described in this review with various components, sizes, dimensions, working media, and modes that can be summarized into story flow of this review article.

In this short section, several typical examples of self-assembled materials with different aspects are introduced as an outline of this review (Figure 2). As well-considered self-assembled objects, formations of one-dimensional fibres and tubes from organic unit molecules are discussed [107–109]. Shimizu and co-workers have successfully accomplished rational preparation of discrete, hollow cylindrical nanoarchitectures from organic unit components, so-called organic nanotubes [110]. Continuous research histories over 30 years have revealed that organic nanotubes can be formed from various organic components including amphiphiles, amino acids, bile acids, carbohydrates, nucleotides, functional dyes, fused aromatics, carbon allotropes, heterocycles, peptides, porphyrins, and other π-conjugated molecules. Although many examples have been presented, there are still remaining issues to be addressed in nanotubes self-assembly research, including (1) purity of morphology between tubular and non-tubular objects such as helical coils, helical twists, non-closed curved sheets, and spherical vesicles or micelles, (2) control of open/close morphologies such as open helical fibre and closed cylinder morphologies, and challenges for scaling-up of experimental examples to the mass production even in ton-scale. Consideration on the latter issues can be applied to many kinds of self-assembled objects not limited to nanotubes.

Because of high selection capability of the component molecules/materials and inclusion of functional groups, materials nanoarchitectonics with self-assembly strategy would be a powerful approach to create functional nanostructures. Ajayaghosh and co-workers have investigated π-conjugated nanostructured materials including specific functional groups such as borondipyrromethene (Bodipy) dyes [111]. Hard tasks for synthesis of the Bodipy derivatives as structural units for their self-assembly are not usually required, giving materials with promising photo-properties including high molar absorptivity, fluorescence quantum yield, and excellent photo-stability. Materials synthesized through their self-assembly are expected to be applied for energy transfer cassettes, liquid crystalline materials, and for bioimaging/labelling usages.

For site-selective modification of inorganic nanostructures, Lvov and co-workers demonstrated site-selective modifications of clay nanotube through self-assembly-type nanoarchitectonics using Schiff base binding [112]. Clay nanotubes are composed of rolling aluminosilicate sheets with diameter of ca. 50 nm, a lumen of 15 nm, and length ca. 1 μm [113,114]. Ligand modification at walls leads to site specific immobilization of heavy metal ions such as Ru^3+^ ion. A similar strategy can be applied to the other heavy metal ions (Ag, Rh, Pt, and Co ions).

Self-assembly processes can be usually conducted under ambient and mild conditions, which makes the processes appropriate for nanoarchitectonics of biological materials [115–118]. Matsuura demonstrated usefulness of carbohydrate-modified DNA and lectin for gene expression regulation, and their assembled system. In his recent review article [119], self-assembly processes of rationally designed biomolecules such as DNAs and peptides were discussed as effective strategies for the developments of drug carriers, nanobio templates, and scaffolds with ligand displaying capability. These strategies are expected to be useful for preparation of vaccines and artificial dynamic nanobiomachines.

In their recent review article, Sawada and Serizawa discuss the use of M13 phage, one of the filamentous viruses, as soft matter units [120]. Upon genetic engineering, M13 phage is originally used as a scaffold for display peptides or proteins on its surface. Self-assembly processes for their conversion to soft materials such as liquid crystalline forms would be beneficial for sensors and biomedical applications.

Self-assembly processes are highly influenced by the surrounding media. Ichikawa and co-workers reported lyotropic liquid crystalline behaviours of amphiphiles in dication-type amino acid ionic liquids [121]. Nature of medium molecules significantly affects critical concentrations of lyotropic liquid-crystalline phase. Bridging their cations and/or introducing aromaticity into their anions reduces the critical concentrations upon promotion of self-assembly of the components. Interfaces often become key media for self-assembly processes because freedom of motions of components and interactions between the components are significantly altered at interfaces [122]. For example, much attention has been paid to the formation of two-dimensional materials, mainly through metal–ligand complexation, which typically results in the formation of metal–organic (porous coordination polymer) nanosheets and can be regarded as interfacial self-assembling nanoarchitectonics [123,124]. Sakamoto reviewed efforts on preparation of low-dimensional coordination nanowires and nanosheets separable as single strands and pieces using dipyrrin metal complexes [125]. Examples in the review highlight the importance of self-assembly processes at selected media for conversion from molecular sciences to low-dimensional materials nanoarchitectonics.

Langmuir monolayers on water would be powerful media to regulate two-dimensional self-assembly [126–128]. Krafft and co-workers summarized importance of molecular designs at Langmuir monolayers of molecules covalently linking the antagonist fluorocarbon and hydrocarbon segments [129]. Nanodomain formations together with regulation of the rheological and other properties of the monolayers through adjunction of diblock designs have been discussed. Such two-dimensional nanoarchitectonic organization in two-dimensional colloidal systems would be applicable in nanoelectronics, photonics, and sensing technologies.

Note that unexplored research issues still remain in self-assembly sciences. Theoretical investigations together with experimental demonstrations have been made for further understanding on the regulations and controls of self-assembly processes and resulting complexes. Hiraoka and co-workers have been systematically investigating the importance of complementary hydrophobic molecular surfaces for precise molecular association to form discrete self-assembled complexes upon quantitative analysis of self-assembly process [130]. Even though the nature of interaction is weak, precise design of the contacting surfaces can create strong and distinct molecular self-assembly structures like the dovetail joint in woodworking. This may be a nice example of nanolevel architectonics, nanoarchitectonics.

Recently, self-assembly processes have been re-evaluated for their dynamics. Formation of supramolecular polymers [131–133], polymeric structures upon non-covalent associations, has been interpreted by dynamic mechanisms, as seen in proposal of living-polymer-like regulations of supramolecular polymers by Sugiyasu, Takeuchi, and co-workers [134]. Dhiman and George summarized their views on temporally controlled supramolecular polymerization [135]. Two dynamic regulations, living and transient supramolecular polymerization, require controls of their length and dispersity and temporally regulated switching of structural and functional states, respectively. Passways into non-equilibrium non-dissipative, kinetic and metastable traps are important issues in these regulations. Non-fuel oscillation systems and fuel-supplied systems are both possible. In recent report, George and co-workers proposed adenosine-phosphate-fuelled systems for temporally programmed supramolecular polymers [136]. Helical assemblies of naphthalene diimide derivative with two zinc(II) dipicolylethylenediamine moities as phosphate receptors can be regulated by adenosine-phosphate upon singular and tandem actions of the related enzymes. This can be a bio-inspired approach for self-assembly process with possible adaptivity and dynamism.

## 3. Recent progresses of self-assembly in three-dimensional media

Following the above-mentioned brief summary of recent trends of self-assembly research, various topics of self-assembly systems are explained below according to certain classifications. In the first category, recent examples of self-assembly systems in three-dimensional media are described.

### 3.1. From small molecular units

#### 3.1.1. Various assemblies from small molecular units

**Figure 3.**
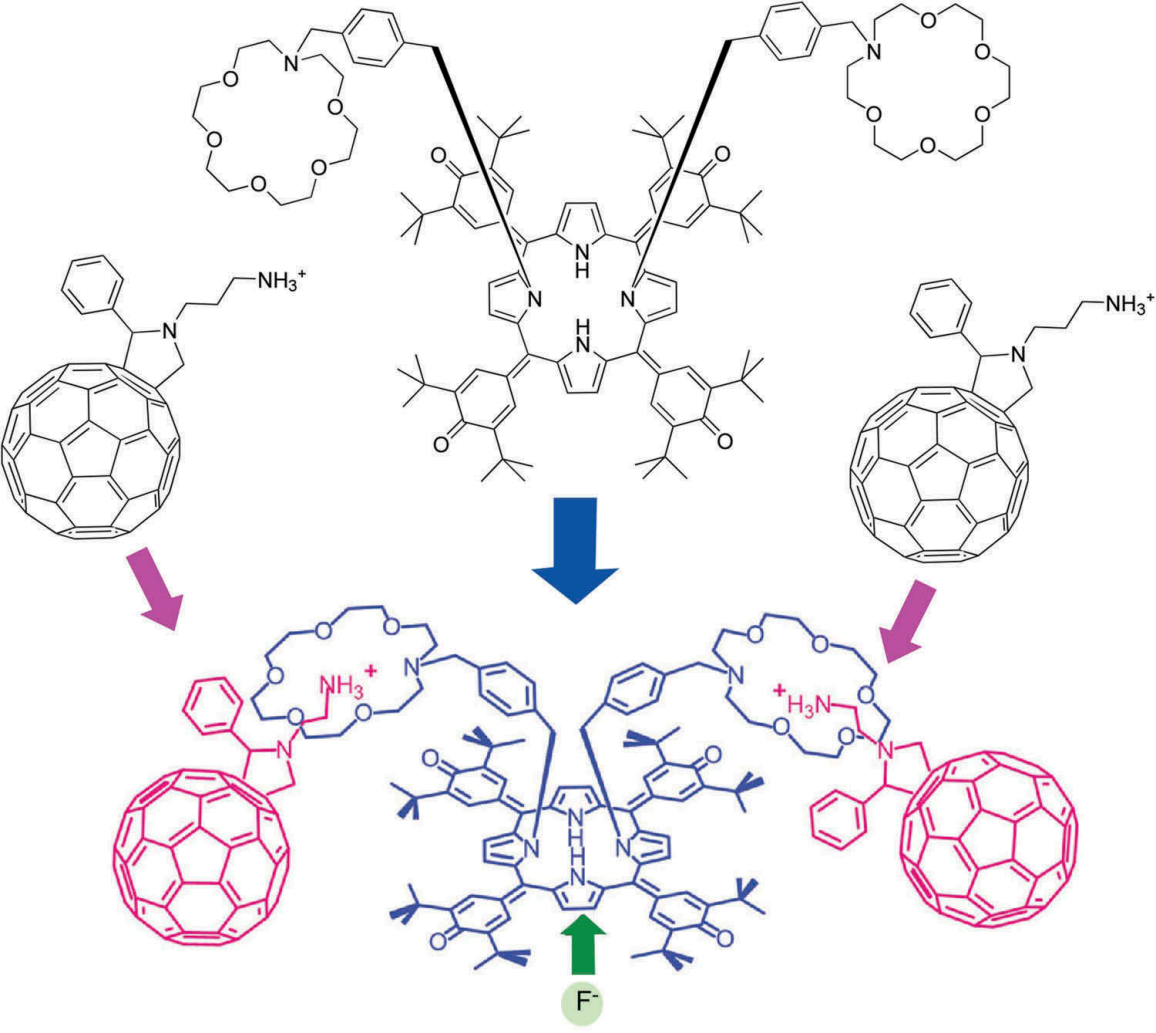
Self-assembled molecular complex where photoinduced charge separation is achieved through fluoride-ion binding.

Self-assembly is a highly versatile methodology to nanoarchitect functional systems from various small units. Sizes of the nanoarchitected systems vary from molecular-level complex to material-size objects. As a small self-assembled functional unit, Hill, D’Souza, and co-workers reported self-assembled molecular complex where photoinduced charge separation is achieved through fluoride-ion binding (Figure 3) [137]. In this molecular complex, bis-crown ether-oxoporphyrinogen is complexed with C_60_ alkyl ammonium cations. Binding of fluoride anion to the oxoporphyrinogen core through hydrogen bonding efficiently promotes the ultrafast charge separation from the singlet excited state of oxoporphyrinogen to C_60_. The charge separation can be maintained through conversion of electron-accepting oxoporphyrinogen unit to a donor by binding with fluoride anion. This nanoarchitected complex can act as a molecular transistor.

Similarly, self-assembled structures often provide unusual physical effects such as regulation of electronic properties. Intramolecular structure of a single molecule can be developed into intermolecular holes through self-assembly as seen in an example demonstrated by Rathore and co-workers [138]. Two-dimensional assembly of pillarene cation radical enables each pillarene to have a fractional formal charge of +1.5 in its crystal lattice. This situation results in long-range charge transport and separation, which would be useful for designing solid-state photovoltaic devices.

Cook and co-workers prepared a self-assembled cofacial cobalt porphyrin prism that acted as a catalyst for the oxygen reduction reaction [139]. The cobalt porphyrin prism having two Co(II) tetra(meso-4-pyridyl)-porphyrinate units and four arene-Ru clips through one self-assembly step without any chromatographic purification processes exhibited an enhanced turnover frequency and successful catalysis for oxygen reduction to hydrogen peroxide almost exclusively (90%). This example presented is advantageous in well-designed self-assembly strategy to create polynuclear catalysts.

Dynamic conformational changes upon self-assembly can tune chiral sensitivity around transition metal complexes as demonstrated by Shinoda and co-workers [140]. In this work, pH-responsive Cotton effects of circular dichroism in the d–d transition bands of assembly of 1,4,7-triazacyclononane with three cholesteryl ester arms were considerably enhanced because of steric interaction between neighbouring complexes. Unexpected mutual influence among the assembled complex species sensitively alters the electronic properties of the molecules.

**Figure 4.**
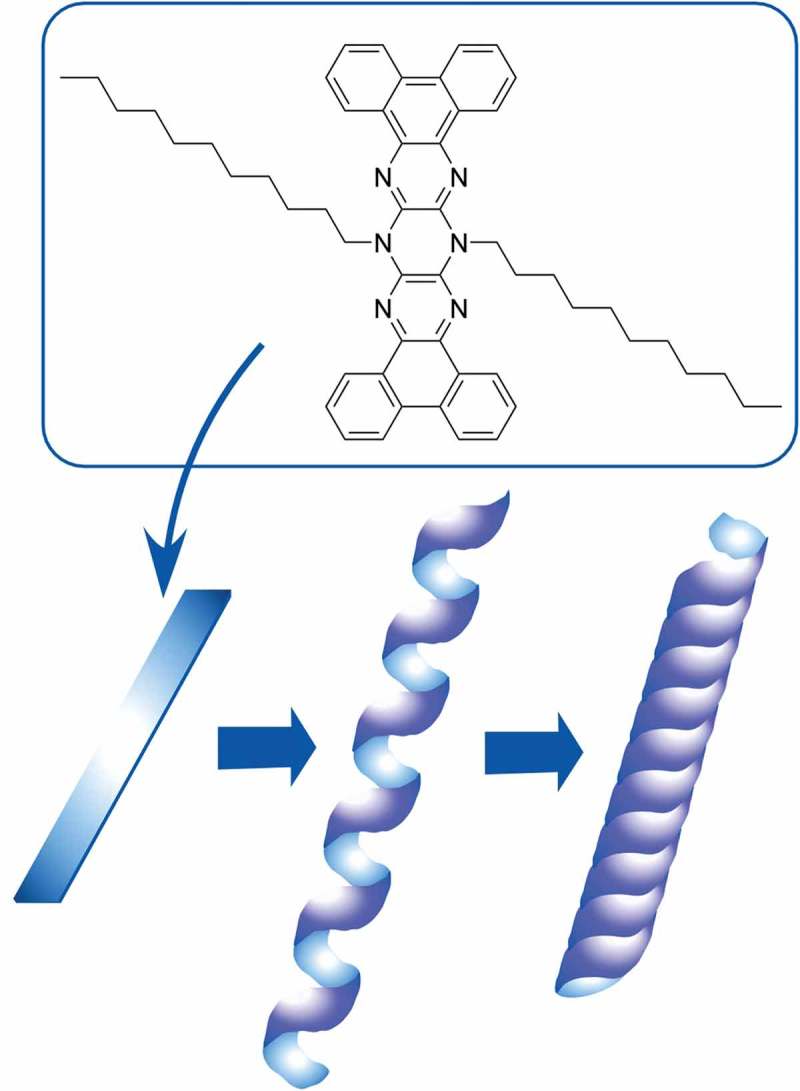
Pyrazinacene nanotube formed with a pentacene derivative,6,13-bis(1-*n*-dodecyl)-[*a,c,l,n*]-tetrabenzo-5,6,7,12,13,14-hexaazapentacene as a molecular unit.

Generally speaking, self-assembly of small molecular units is useful strategy for formation of structure-controlled materials. Hill and co-workers demonstrated well-controlled preparation of pyrazinacene nanotubes using a pentacene derivative, 6,13-bis(1-*n*-dodecyl)-[*a,c,l,n*]-tetrabenzo-5,6,7,12,13,14-hexaazapentacene as a small molecular unit (Figure 4) [141]. In this self-assembly process, a 130-nm-wide two-dimensional tape object was initially formed and then converted into a hollow cylindrical form (150–200 nm diameter and more than 10 µm in length) through helical twisting of the initially formed tape. Chromophore parts would form J-aggregates. Self-assembly of this nitrogen-rich pentacene analogue is expected to show properties significantly different from the parent non-aggregated unit molecules.

Nakanishi et al. successfully prepared hierarchic nanoarchitectures through self-assembly of C_60_ derivatives with different numbers of long hydrocarbon chains and semiperfluoro-alkyl tails [142]. Three-dimensional microparticles with nanoflakes at the external surfaces or microparticles with many plate-like units were obtained by the self-assembly of hydrocarbon-branched C_60_ derivatives. On the other hand, the C_60_ derivative with semiperfluoro-alkyl chains provides surfaces with good water-repellence from its diethoxyethane solution. The assembled structures are expected to be used for non-wetting, low adhesion, and self-cleaning surfaces. As materials transcription from the self-assembled structures, Nakanishi et al. also reported preparation of nanoflake-metal surfaces through sputtering various metals such as Au, Pt, Ti, and Ni on the self-assembled nanoflake objects of the C_60_ derivatives followed by removal of the C_60_ components by solvent extraction [143]. Especially, Au nanoflake structures exhibited excellent capabilities as substrates for the surface-enhanced Raman spectroscopy.

Besenius and co-workers demonstrated step-wise self-assembly of Au(I)-metallopeptide in water [144]. This molecular unit first kinetically assembled into metastable nanorods at room temperature and converted into fibrils upon heating resulting in the formation of dimeric bundles. Balances between strong associative supramolecular interaction in water and repulsive forces can create conditions for non-equilibrium self-assembly processes.

Photo-controllable formation and deformation of supramolecular polymer was reported by Chen, Wang, and co-workers [145], who used host–guest interactions between macrocyclic cucurbit[8]uril and monomer unit with 9,10-dialkoxyanthracene as an electron-rich central core and monocharged 4,4-bipyridinium terminals. Intramolecular donor–acceptor interaction of the monomer unit was photochemically stabilized, but the photolysis of 9,10-dialkoxyanthracenes was enhanced upon complexation with the cucurbit[8]uril. The formed linear supramolecular polymers have photodegradable nature. Such strategy for self-assembled supramolecular polymers with regulation of photo-reactivity would be useful for applications such as photodynamic therapy and photo degradation of materials.

**Figure 5.**
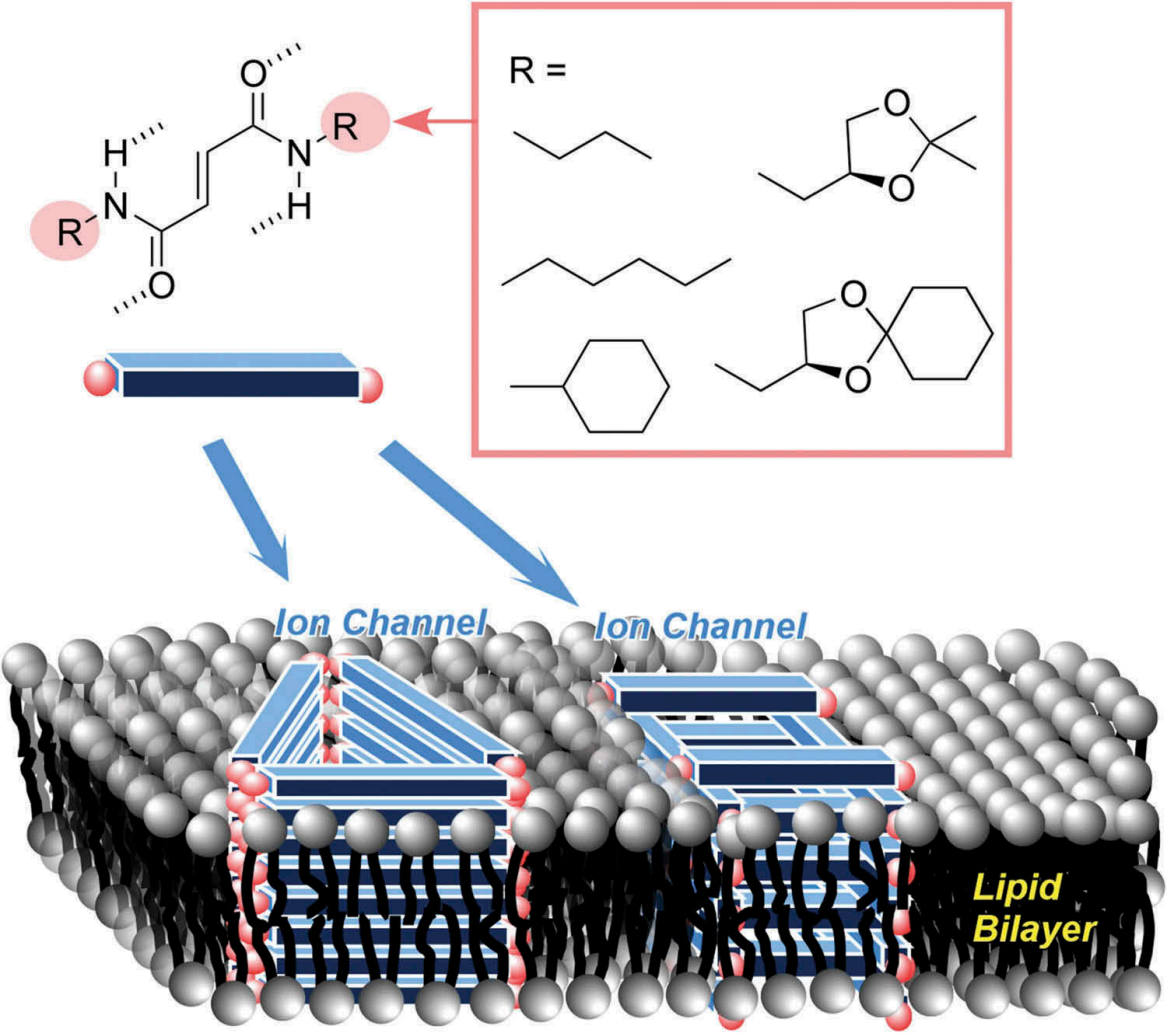
Transmembrane chloride channel formed through self-assembly of small-molecule fumaramides.

Nanoarchitectonics upon self-assembly process to promote biomimetic and bio-inspired functions is also attractive research target [146,147]. For example, Talukdar and co-workers reported formation of transmembrane chloride channel through self-assembly of small-molecule fumaramides (Figure 5) [148]. Fumaramides and the corresponding maleamides with various *N*-terminal hydrophobic groups were synthesized and self-assembled into transmembrane channel structures within lipid bilayer membranes. Selective transmembrane transport properties of Cl ions were confirmed by planar bilayer conductance measurements. Cell-membrane-like assembled structures such as vesicles and structured micelles can be nanoarchitected from various small molecules. As an interesting example, Knight, Hawker, and co-workers reported morphologically shifting spherical assemblies upon metal coordination to assemblies of hybrid peptide–polymer conjugates [149]. Aggregated micellar particles were formed with Zn(II), Co(II), and Cu(II) ions. The presence of Mn(II) resulted in multilamellar vesicles, while formation of micelles was induced by Ni(II) and Cd(II) species.

#### 3.1.2. Coordination-based assemblies from small molecular units

As compared with some molecular interactions such as hydrophobic effects and electrostatic interaction, coordination provides specific and strict molecular pairing. Self-assembly processes based on coordination interactions play unique roles in self-assembly science. For example, Severin and co-workers systematically investigated effects of size and geometry of the ligand on the self-assembled structures of Pt-based coordination self-assembly [150]. In some cases, formation of barrel- or gyrobifastigium-like structures with a molecular weight of more than 23 kDa and a diameter of 4.5 nm can be assembled. Generally speaking, coordination interaction promotes both continuous regular structures, such as metal–organic frameworks and coordination polymers, and isolated objects with strict size and shape including molecular capsules and coordination cages. Especially, the latter characteristic is useful for precise structural regulations and structural shifts/tuning.

Jia, Wu, and co-workers reported structural shifts of coordination capsules between A_4_L_6_ tetrahedral cage and A_2_L_3_ triple helicate [151], where A and L represent anion and ligand, respectively, upon coordination self-assembly from phosphate anion and a C_2_-symmetric bis–bis(urea) ligand. Guest inclusion stabilizes the tetrahedral cage structure and removal of guest convert the cage to triple helicate. Interestingly, peripheral templating had effect to unusually assemble empty cage, which can be utilized for capture and release of biologically important guest molecules including choline and acetylcholine.

Jin and co-workers demonstrated structural conversion between stacked arrays and Borromean rings using a dinuclear naphthalenediimide-based acceptor and linear bipyridyl ligands [152]. The former dinuclear naphthalenediimide unit possesses important features of dynamic coordination modes for flexible structural shifts and electron-deficient aromatic flat surface for donor–acceptor π-interaction. Inclusion of donor guest such as pyrene molecules resulted in the formation of discrete aromatic stacking arrays with vertical π-conjugations. In addition, Borromean ring structures were architected with different guests.

Coordination-based self-assembled structures can precisely decide the mutual dispositions of molecular components, which is sometimes advantageous for regulation of reaction as reported in controlled solid state reaction by MacGillivray and co-workers [153]. In their system, imine bond formation for pyridyl to the alkene *trans*-cinnamaldehyde was conducted first, and the formed pyridyl-functionalized alkene molecules were positioned for appropriate geometry of the solid state for an intermolecular [2 + 2] photodimerization upon metal–organic assembly with Ag(I) ions. Through this one-dimensional coordination polymer route, cycloaddition reaction to pyridyl-functionalized cyclobutane can be stereo-selectively achieved with quantitative yield. Precise structure formation upon coordination-based self-assembly can be used for selective encapsulation of guest molecules. Selective capture of C_60_-indene or C_60_-anthracene bisadducts by cubic cage assembled from 4-fold-symmetric porphyrins with Fe(II) or Zn(II) was reported by Nitschke and co-workers [154]. Well-designed cage structure also becomes appropriate media for the reaction of C_60_ and anthracene to the bisadducts.

Inclusion of lanthanide elements to the coordination self-assembled structures has potentials for various applications such as sensing, label-imaging, magnetic resonance imaging, catalysis, and magnetic refrigeration. Sun and co-workers demonstrated structural switching of lanthanide-including coordination self-assembled cages [155]. Variation of ligand structures with different distances between the two chelating arms controlled the assembled structures. Under borderline conditions, structural switching between the helicate and tetrahedral structures was observed. Some luminescent lanthanide cubes worked as excellent turn-off sensors for the detection of explosives molecules such as picric acid at ppb level.

Zhou, Li, and co-workers prepared coordination cages with defined chirality and inner space [156]. Chiral cobalt-imidazolate cages assembled from 72 sub-components exhibited tetartoidal structures including tetragons, pentagons, and dodecahedrons, with spontaneous resolution of racemic tetartoidal cages (Δ and Λ) into a conglomerate of homochiral crystals. Chiral induction of l- and d-enantiomers of menthol resulted in both homochiral Δ and Λ tetartoidal cages, respectively. The formed structures are expected to be used for various applications including asymmetrical catalysis and enantioselective separation.

Preparation of graphene-like honeycomb single and multilayer structures through self-assembly was demonstrated by Hou et al. [157]. Fan-shaped building blocks were first synthesized by covalent linkages between one polyoxometalate and four polyhedral oligomeric silsesquioxanes, which can be self-assembled into two-dimensional honeycomb superlattice as mesoscale analogue of graphene. The formed two-dimensional materials possess huge surface area and porosity that are advantageous for high accessibility to active sites and excellent chemical affinity. Potential applications are expected such as specific catalysis, sensors, electrochemical applications, various membrane functions, two-dimensional heterojunctions, templates for nanomaterials synthesis, and nanoreactors.

### 3.2. From polymers

**Figure 6.**
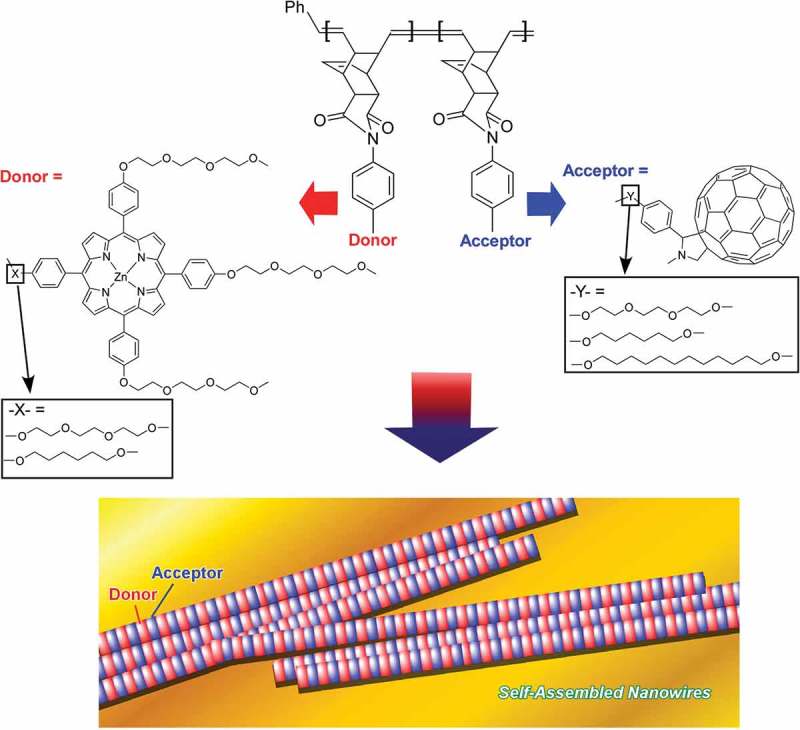
Stacked p–n heterojunction arrays of nanowires of nanosized acceptor/donor domains formed through self-assembly of structure-designed block copolymers.

Polymer materials have various unique characteristics as the components of self-assembly [158–161]. Molecular segments with attractive and/or repulsive interactions can be co-embedded within one polymer chain possibly with various structural motifs such as linear block, branched, grafted, and dendric structures. The structural variety of the component polymers results in a vast range of structures and functions after their self-assembly. In addition, characteristic robust nature of the polymer components is advantageous for practical applications. Therefore, self-assembly of block copolymers is often used for nanoarchitectonics fabrication of functional structures. For example, Charvet et al. reported fabrication of stacked p–n heterojunction arrays of nanowires of nanosized acceptor/donor domains through the self-assembly of structure-designed block copolymers (Figure 6) [162]. Block copolymers having C_60_ fullerene (acceptor part) and tetraphenylporphinatozinc(II) (donor part) synthesized by living ring-opening metathesis polymerization were self-assembled into one-dimensional nanowires with phase-segregated structures with ca. 5.5 nm periodicity. Self-assembly nanoarchitectonics to form this zebra-stripe-like nanowires can be accomplished only by drop-casting of the block copolymer solutions onto a solid substrate such as highly oriented pyrolytic graphite or mica. The prepared films including self-assembled nanowire arrays showed electrical conductivities (up to 6.4 × 10^−4^ cm^2^ V^−1^ s^−1^) and high charge carrier mobility (ca. 0.26 cm^2^ V^−1^ s^−1^). In addition, the fabricated films exhibited repeatable photocurrent on/off switching in response to the white light irradiation.

Regulation of shapes and morphologies of polymer materials is an important subject regarding the control of their functions such as optical properties. Such regulation can be sometimes achieved by self-assembly of well-designed block polymers within the confined environment. Kim and co-workers reported nanoarchitectonics fabrications to provide assembled structures of polystyrene-*b*-polybutadiene block-copolymer in prolate ellipsoids, onion-like spheres, and oblate ellipsoids through evaporation of the polymer emulsions [163]. Relatively fast evaporation rate resulted in the formation of prolate ellipsoids with axial lamellar stripes. Under this condition, large difference of solvent (toluene) diffusion rates between polystyrene and polybutadiene moieties caused the orientation of lamellae perpendicular to the particle surface. This situation is suitable for the formation of the ellipsoid with axially stacked lamellae. Slow evaporation rate resulted in the formation of onion-like particles that have lamellae structures with an outermost layer of polybutadiene. In addition, several parameters for the morphological transition and the aspect ratios in the ellipsoids have dependence on molecular weight of the block copolymers.

Structures of unit polymers for self-assembly strategies are not limited to linear ones. Self-assembly of hyperbranched polymers is also attractive subject as seen in research work on self-assembly of hyperbranched poly(ionic liquid)s with controlled peripheral amphiphilicity reported by Tsukruk and co-workers [164]. They investigated self-assembly behaviours of amphiphilic hyperbranched poly(ionic liquid) molecules with various number ratio of peripheral hydrophobic arms to the hydrophilic ionic groups in water and at the air–water interface. The morphology of the assembly formed can be regulated depending on the balance of intermolecular interactions with the ionic liquid components at the inner shell of the hyperbranched polymers. As the number of hydrophobic tails increases from 0 to 24 arms, larger and highly charged micellar aggregates tend to form in aqueous solution, resulting in the increase of size from 100 up to 240 nm. According to similar aggregation mechanism to unimolecular micelles, higher level micelles are probably formed hierarchically from hyperbranched poly(ionic liquid) micelles. This nanoarchitectonics strategy upon amphiphilicity adjustment of the component polymer to tune materials morphology is expected to be useful for materials developments for various applications such as energy storage.

**Figure 7.**
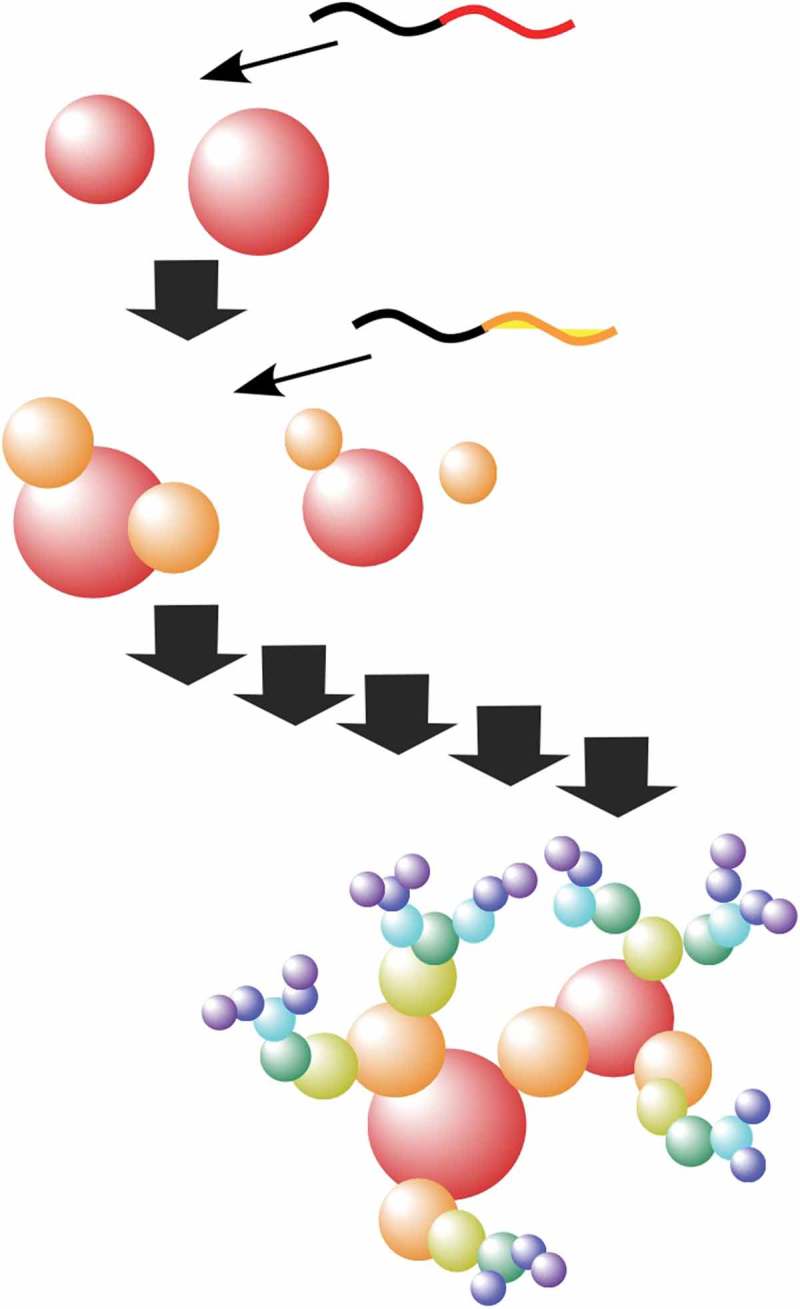
Synthesis of polymer fractal nanostructures upon controls of polymerization and self-assembly kinetics in solution media.

As one of the useful materials with high surface area for various usages including coatings, catalysis, sensing, electronic applications, and biomedical uses, fractal nanostructure of polymeric materials through self-assembly is an attractive object. Choi and co-workers proposed a novel method upon controls of polymerization and self-assembly kinetics to realize a one-shot synthesis of polymer fractal nanostructures in solution media (Figure 7) [165]. Based on higher reactivity of one monomer than the other, a diblock copolymer microstructure was first formed, and then the second insoluble block components containing poly(*p*-phenylenevinylene) self-assembled, resulting in *in situ* nanoparticlization of conjugated polymers. Tuning polymerization conditions including monomer concentration and additives can regulate the branching patterns of the fractal polymer nanoarchitectonics. The proposed strategy upon shifting polymerization kinetics and equilibrium of polymer self-assembly would be useful for tuning of nanoarchitectonics structures.

Grubbs and co-workers reported nanoarchitectonics strategy for the self-assembly of polymers having tailored distributions of grafting sites by grafting in ring-opening metathesis polymerization [166]. Sequences of polymer backbone and side chain distribution were regulated by copolymerization of an *ω*-norbornenyl macromonomer and norbornenyl comonomers as diluents including polystyrene, poly(d,l-lactide), and polydimethylsiloxane macromonomers. On the basis of differences of reactivity ratios between macromonomer and diluent controls, the backbone sequences as blocky, gradient, or random as well as graft diblock polymer structures with tapered, uniform, and inverse-tapered molecular shapes. Self-assembly of these polymers creates nanoarchitectonic structures with different lamellar periods and domain thicknesses. Because of general applicability of the proposed method, it can be applied for various types of polymer materials and their assemblies possibly for future extensive customization.

High capability in flexible structural shifts of polymer and oligomer self-assemblies can be used for various applications such as stimuli-responsive functions. Chu and Ravoo demonstrated preparation of hierarchical supramolecular hydrogels through peptide self-assembly with photo-controlled guest release functions, using Fmoc-RGDS peptide functionalized with photo-switchable arylazopyrazole [167]. Self-assembly of the designed peptide molecules resulted in the formation of hydrogel materials that can be used for light-triggered materials release. Releasable substances are not limited to conventional small molecules, and vesicles can be used as large substance payload, suggesting a wide range of usability for photo-controlled release of drugs and biomaterials.

Site-specific arrangements of responsive polymer self-assembly can be used for macroscopic actions as seen in actuator functions of surface microscopic gel arrays reported by Morin and co-workers [168]. They proposed a surface moulding method for the controlled gel arrangement in which geometry of liquid prepolymer microdroplets was manipulated on the chemically patterned substrate and photo-initiated cross-linking resulted in morphology-preserved arrangement hydrogel microstructures. This method can be regarded as soft lithographic method to fabricate microscopic arrangement of polymer self-assembled materials with wide ranges of geometrical and functional varieties. The formed arrays on the substrate can be used as stimuli-responsive actuators and their motions can be programmed based on the variety of their geometrical and functional features with good potential impacts in engineering, materials science, chemistry, and biology.

### 3.3. From inorganic materials

Application of self-assembly concepts is not limited to organic substances. Nanoarchitectonics strategy upon self-assembly is applicable to systems with metallic and inorganic substances in which self-assembly of inorganic materials themselves and hybrid assemblies between organic and inorganic substances can be considered [169–177]. As self-assembly of functional parts on gold surfaces for sensing applications, Prins and co-workers demonstrated fluorescence sensing systems through nanoarchitectonics on Au nanoparticles [178]. On the surface of Au nanoparticles, functional moieties such as 1,4,7-triazacyclononane Zn(II) complex and a 1,4,7,10-tetraazacyclododecane Zn(II) complex were immobilized together with fluorogenic probe molecules. Upon selective binding of guest molecules to the complex part, release of the fluorogenic probe molecules from the surface of nanoparticles to solution resulted in fluorescent signals. This strategy can be applied in versatile combination of recognition and fluorescent functional units, which possibly enables to nanoarchitect various sensing systems for many kinds of chemical inputs. Harlé, Fujiwara, and co-workers immobilized malachite green derivatives as a self-assembled monolayer (SAM) on TiO_2_ [179]. The malachite green derivatives were characterized at negative excited state redox levels for more appropriate in use of dye-sensitized solar cell applications.

Qin et al. prepared highly symmetric lanthanide clusters as inorganic examples of topological self-assembly [180]. A series of high-symmetry Gdm(III) polyhedra, Gd20, Gd32, Gd50, and Gd60, were prepared through self-assembly of polymetallic fragments in different polygon structures. Detailed analyses based on magnetization measurements related Gd-O-Gd angles and the cluster bands, which could be used as fingerprints to specify these cages.

Regulation of multidimensional self-assembly of quantum-sized materials is crucial for the development of advanced devices. Ji et al. demonstrated fabrication of Au nanoparticle superstructures based on ionic-liquid-like alkyl imidazolium-mediated mechanisms [181]. Using imidazolium with decyl chain as nanoparticle stabilizer, Au nanoparticles are self-assembled into a core-shell spherical superstructure through assembly of imidazolium molecules during ligand exchange process. The formed superstructures have possibilities for various applications including sensing, catalysis, biotherapy, and photonics. Acharya and co-workers similarly reported controlled self-assembly of Au nanoparticles into one-dimensional, two-dimensional, and three-dimensional structures through careful selection of ligands [182]. Such tuneable multidimensional self-assembly of quantum-size objects would be beneficial for optoelectronic devices with the tuneable optical properties.

**Figure 8.**
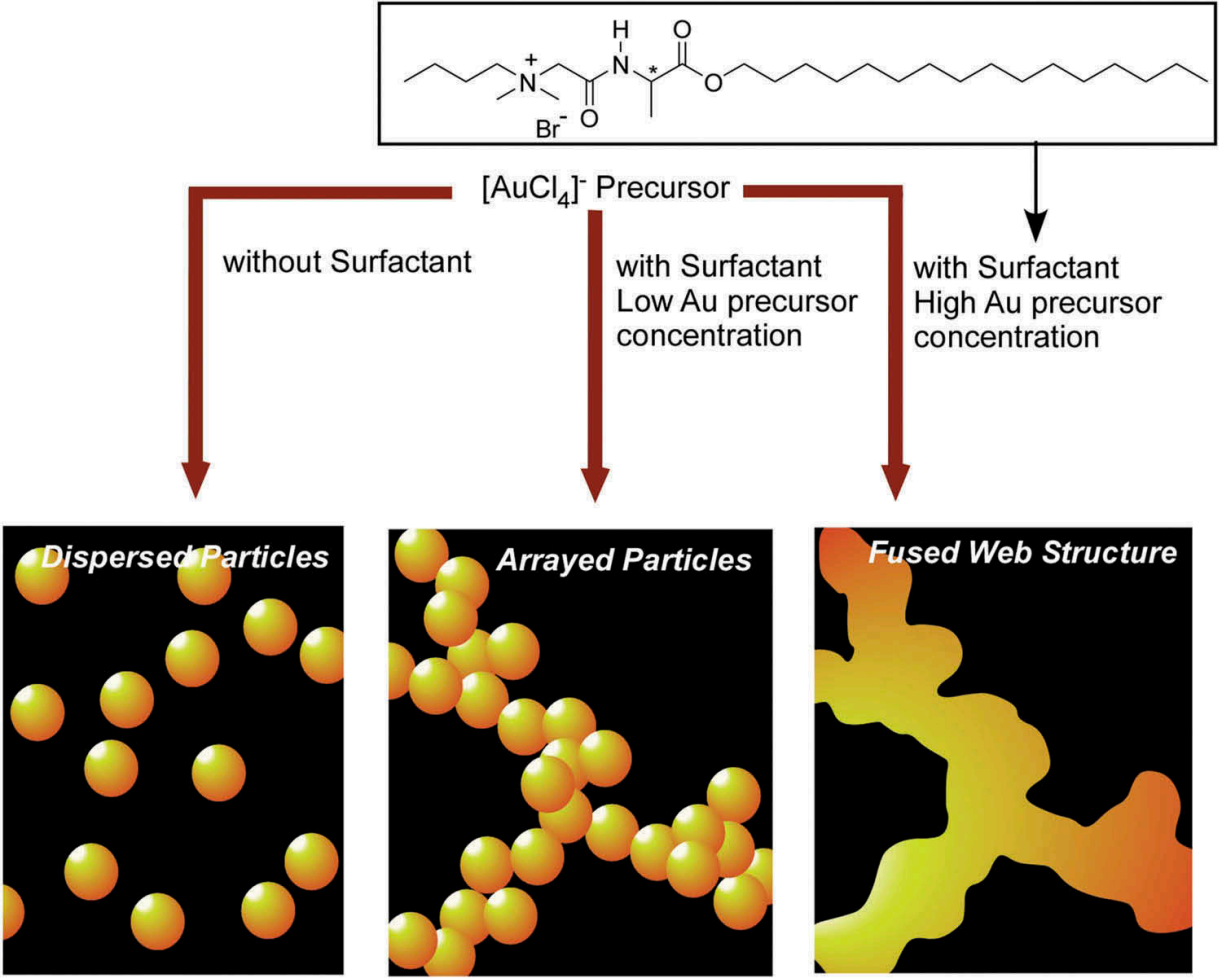
Two-dimensional nanoweb structure of gold prepared through web-like supramolecular self-assembly of surfactant followed by *in situ* reduction of the gold precursors.

Two-dimensional nanoweb structure of gold can also be prepared through web-like supramolecular self-assembly of surfactant followed by *in situ* reduction of the gold precursors (Figure 8) [183]. Initially, surfactant assemblies were stabilized by hydrogen bonding between surfactants, which work as template for room temperature fusion (cold fusion) for gold nanostructures. Because gold surfaces are good medium for immobilization of organic functional groups through SAM formation, the formed two-dimensional Au nanoweb structure can be applied in the integration of organic functions in two-dimensional plane. Huang et al. utilized self-assembling behaviours of gold nanostructures for fingerprint detection [184]. For this purpose, gold nanoclusters protected with bovine serum albumin were efficiently synthesized with the aid of ultrasonic-microwave heating. The formed Au nanoclusters with mean diameter of ca. 3.3 nm have a high quantum yield of 7.1% and can be applied in the detection of latent finger-marks.

**Figure 9.**
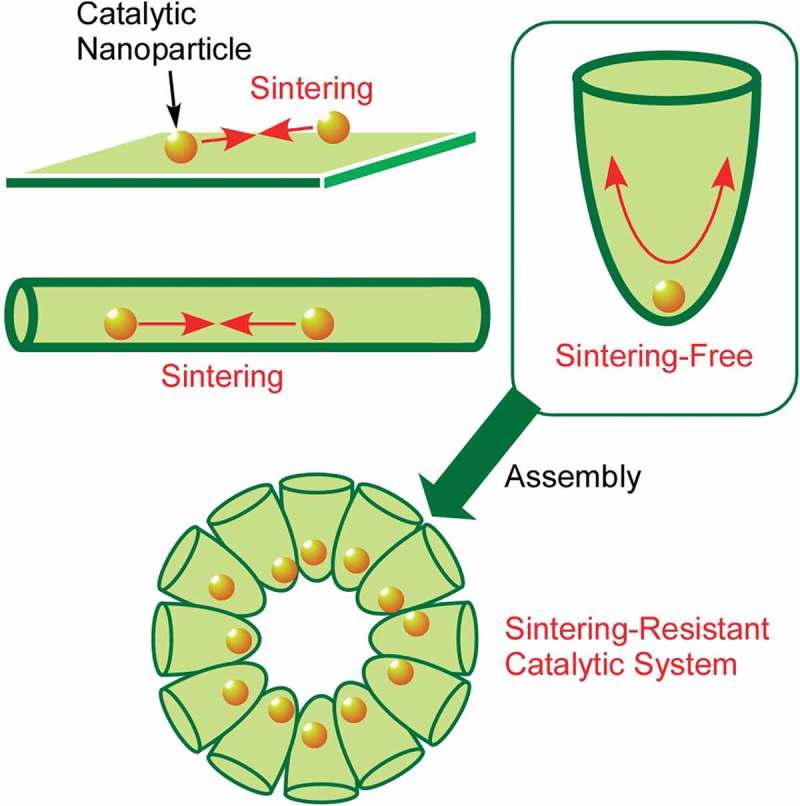
Sintering-resistant nanoparticle systems through confinement of catalytic Pt nanoparticles within compartments prepared with self-assembled silica nanostructures.

Control of nanoparticle assembly is sometimes crucial for regulation of catalyst activity because aggregation and sintering of catalytic nanoparticles seriously affect their activities. Liu et al. presented a novel strategy for sintering-resistant nanoparticle systems through confinement of catalytic Pt nanoparticles within compartments prepared with self-assembled silica nanostructures (Figure 9) [185]. This strategy maximizes the travelling distance between neighbouring Pt nanoparticles within wide-mouthed silica compartments. Access of reaction substances to the Pt catalysts is ensured and probability of the nanoparticle sintering is minimized. In fact, the entrapped Pt catalyst exhibited much higher CO oxidation activity than the nanoparticles immobilized in the other nanostructures such as mesoporous silica. The proposed methodology for confining the functional nanoparticles in open-access compartments with sintering-resistance capability would be useful for various kinds of sustained performance of functional nanostructures.

Nakata et al. reported synthesis of self-assembled fluorescent clusters of seminaphthorhodafluor derivative that were protected by a photo-removable *o*-nitrobenzyl group [186]. The synthesized nanoclusters self-assembled into a colourless and nonfluorescent assemblies, and UV irradiation converted them into fluorescent structures. Enhanced pH-responsive fluorescence can be observed both in solution and HeLa cell. Therefore, the assembled clusters would be useful for a dual-emissive ratiometric fluorescent pH probe both for chemical and biological applications.

Processes based on dissipative self-assembly are well seen in natural systems, but such out-of-equilibrium assembly has not been fully explored in artificial systems. As rare example, Sawczyk and Klajn reported out-of-equilibrium assembly of Au nanoparticles [187]. In the demonstrated system, Au(III) ions are reduced to attach onto the existing Au nanoparticles seeds accompanying with increase of surfactant concentration. This process induces the formation of surfactant bilayers, resulting in nanoparticle assembly. According to shifts of surfactant concentration, the formed aggregates of Au nanoparticles gradually disassemble. The lifetimes of this out-of-equilibrium self-assembly can be regulated by the size of the constituent Au nanoparticles. Self-assembly of inorganic structures into complex forms is still a challenging subject.

Kotov and co-workers demonstrated the formation of mesoscale hedgehogs of FeSe_2_ from puck-like-shaped primary FeSe_2_ nanoparticles with size 2–4 nm as well as formation of monocrystalline nanosheets with thickness of 5.5 nm and lateral dimension of 1000 nm [188]. The latter nanosheet structures are parts of the hedgehog morphology and are rolled to form scrolls at the around cores as needles normal to the core. The core is composed of assembly of the primary nanoparticles and nanoribbons. Concentration of dodecanethiol in solution is an important factor for the assembling route, but the polydispersity of the primary unit is not detrimental for the complexity of the self-assembled structures.

As widely known, self-assembled structures are used as template for the synthesis of various nanostructured materials [189–191]. Yamauchi and co-workers reported fabrication of a series of porous carbon spheres with controlled textural parameters including mesopore diameters, specific surface areas, and particle sizes [192]. This control is achieved by tuning the degree of polymerization of polystyrene in polystyrene-block-poly(ethylene oxide) templates.

Such structure-regulated nanocarbon materials are expected to be useful for various applications such as electrochemical supercapacitors. Magana et al. demonstrated the fabrication of carbon nanofibers with high surface area through nanocasting process from silica nanofibers prepared with self-assembled chromonic liquid crystals templates [193]. Good electrochemical supercapacitance performances were obtained due to randomly oriented graphitic layers. Their long lifetimes were also confirmed through retention of ca. 95% of the capacitance capability after 1000 cycles.

Vinu, Kim, and co-workers reported synthesis of mesoporous materials of cationic 3,4-propylenedioxythiophene-silica [194]. These mesoporous materials were synthesized by combined processes of self-assembly of the silica surfactant and *in situ* polymerization of 3,4-propylenedioxythiophene. This methodology can be applied to various substances to provide various porous materials with high surface area useful in many applications such as sensors, drug delivery systems, fuel cells, and organic devices.

### 3.4. From biomolecules

#### 3.4.1. DNA-based assemblies

Because biological systems are made by self-assembly of various component molecules including lipids, peptides, proteins, nucleic acids, polysaccharides, and so on, these biomolecules can be also good components of self-assembly processes for artificial functional systems [195–199]. One of the typical successful examples would be DNA nanotechnology such as DNA origami in which various structures can be logically nanoarchitected through specific base-paring and programmed DNA sequences [200–202]. Schulman and co-workers demonstrated hierarchic self-assembly of DNA nanotubes in micrometre scale from DNA strands with programmed sequences [203]. Even though the assembled structures expanded into micrometre scales, their geometry can be decided by smaller scale DNA origami junctions. The DNA origami junctions have either of L-, T-, and Y-shape, from which two or three DNA nanotubes in micrometre length can be grown. Relative angles of these micrometre scale tubes reflect the shapes of nanoscale junctions, implying that nanoscale nanoarchitecture can precisely determine the shapes of micrometre objects. This example indicates the high potentials of DNA origami technology to architect two- and three-dimensional structures including circuit-like logical networks and extended materials.

Nanoarchitectonics from mixtures of significantly different shapes has much potentials to logically construct functional structures. Lattice controls through self-assembly of spherical Au nanoparticles and DNA bundle rods have been experimentally and theoretically demonstrated by Gang, Panagiotopoulos, and co-workers [204]. The spherical and rods units are specifically interacted each other via recognition between complementary DNA sequences. Upon increase of nanoparticle diameter with fixed rod length, translons from their disordered phase to the hexagonal close-packed crystal, which is supported by attractions between rods across alternating crystal layers. Further increase of nanoparticle size resulted in the stabilization of face-centred cubic lattice because of rod entropy. These structure controls can be accomplished by appropriate balancing between the energy and entropy of the rods in self-assembling process, and this concept can be extended to assembling units with more complicated shapes for more exotic self-assembled structures.

**Figure 10.**
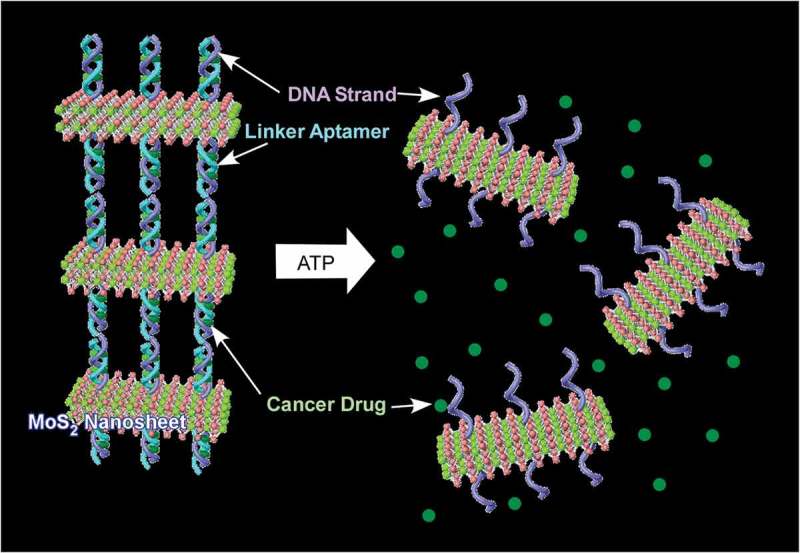
Drug delivery systems controlled by DNA self-assembly and disassembly between MoS_2_ nanosheets.

As functions based on DNA self-assembly, Leong and co-workers demonstrated drug delivery systems controlled by DNA self-assembly and disassembly between MoS_2_ nanosheets (Figure 10) [205]. MoS_2_ nanosheets can be functionalized with DNA oligonucleotides through strong binding of sulphur atom at the terminal of DNA onto the defect vacancies on surface of MoS_2_ nanosheets. The functionalized nanosheets were assembled into layer-by-layer structures with the aid of linker aptamers. Cancer drugs can be carried within the layer-by-layer structures with sufficient protection from external attacks such as lysosome. However, the layer-by-layer structure allows penetration of small molecules. Diffusion of adenosine triphosphate (ATP) into interlayer region causes disassembly and drug release upon strong binding of ATP with the linker aptamers. Therefore, drugs can be released only under ATP-rich conditions. This protection and controlled release strategy can be applied to many situations by changing DNA designs.

#### 3.4.2. Assemblies with proteins and peptides

Although pairing specificity of recognition may be slightly inferior to DNA, peptides and proteins surely have high capability of self-assembly mainly through hydrogen bonding. Accumulation of hydrogen bonding upon well-designed three-dimensional nanoarchitectures often creates surprisingly precise recognition sites [206–209]. Peptides and proteins have been widely used as unit structures of self-assembly nanoarchitectonics. As seen in naturally occurring protein structures, self-assembly of polypeptide segments is useful for nanoarchitecting hierarchical structures. Rotello and co-workers reported extension of unit assembly of recombinant proteins and Au nanoparticles into superstructures with sizes of hundreds of nanometres [210]. In their methods, green fluorescent proteins with a genetically incorporated glutamic acid peptide chain and Au nanoparticles bearing arginine-terminated ligands were used, which can be self-assembled through strong carboxylate–guanidinium interactions. Modified green fluorescent proteins and Au nanoparticles first assembled into granules and further self-assembly resulted in sub-micrometre-level superstructures. Within the hierarchic superstructures, components can be dynamically and spatially reorganized in response to environmental conditions. Such hierarchically assembled materials are expected to be used for various applications such as catalysis, drug delivery, and energy harvesting.

Preparation of protein–metal–organic frameworks (protein–MOFs) was demonstrated by Tezcan and co-workers who could self-assemble the metal ions presented at the vertices of the ferritin nodes in the presence of dihydroxamate linkers in synthesis of protein–MOF structures [211]. The formed ferritin–MOF structures are assigned as body-centred (cubic or tetragonal) lattice arrangements with dynamic nature. This strategy includes high synthetic modularity to possibly generate protein-based crystalline materials. As another type of porous protein materials, Vinu and co-workers successfully synthesized nanoporous cytochrome c films using self-assembled polystyrene spheres as templates [212]. Electrochemical properties of cytochrome c were retained for long time. Combined strategy of self-assembly and template synthesis can be used for preparation of porous materials of various biomolecules possibly including proteins, peptides, polysaccharides, and nucleic acids.

Well-designed peptide segments are also good components for self-assembly. Kimura and co-workers investigated self-assembled morphologies of various types of amphiphilic block polypeptides having aromatic groups at the C-terminal of a Leu-based hydrophobic helical block and actually confirmed self-assemblies into various structures such as distorted sheet, saddle-like sheet, micelle, and so on [213]. Upon heating at 90 °C, transformation from nanotubes to vesicles was also observed. The observed morphologies were much influenced by the presence of aromatic groups at the C-terminal.

Coacervate microdroplets are expected to be used for synthetic cell applications but they have to be stabilized with an appropriate coating. Williams, van Hest, and co-workers proposed the use of biodegradable triblock copolymer (terpolymer) with poly(ethylene glycol), poly-(caprolactone-gradient-trimethylene carbonate), and poly-(glutamic acid) segments [214]. The designed polymers have advantages of electrostatic coacervate anchoring, hydrophilic surface buoyancy, and hydrophobic membrane self-association. Stabilized cell-sized coacervate microdroplets are regarded as hybrid protocells and expected to be used for complex synthetic cell applications.

## 4. Recent progresses of self-assembly at interfaces

Interfaces such as solid surfaces, liquid–liquid interfaces, and gas–liquid interfaces are the media that cause various interesting effects in molecular interactions and self-assembly behaviours as compared with those observed in bulk solution media [215–217]. In the following section, recent research examples on self-assembly at interfaces media are explained. At interfacial two-dimensional environments, molecular motions and orientations are restricted. Interfaces become meeting points from materials from different phases. Heterogeneous dielectric nature of some interfacial media significantly affects molecular interactions [218]. The vast size difference between the macroscopic lateral dimensions and nanoscale thickness allows to couple macroscopic actions and molecular functions [219,220]. Fixation of self-assembled structures on solid surface often become an important step for device fabrication. These characteristics make self-assembly and supramolecular chemistry at interfaces an attractive research subject.

### 4.1. Surface nanostructures (SAM, etc.)

One of the powerful and popular interfacial media for self-assembly nanoarchitectonics is supplied by SAMs and surface-adsorbed films on solid surfaces [221–223]. Self-assembled structures formed on solid surface can be used for device applications and surface nanofabrication. Konishi and Yamaguchi reported nanoarchitectonics of photo-responsive SAM with a 2-nitrobenzyl carbamate moiety and selective modification with Ag nanoparticles and fluorescent dye molecules [224]. The SAM structures were prepared with silane coupling chemistry on thermally surface-oxidized silicon wafers and were photo-patterned by irradiation with soft UV light through a photomask. Ag nanoparticles were site-selectively deposited by spin coating. The photo-deprotected surface with NH_2_ groups was selectively covalently modified with fluorescent dye having a succinimidyl ester moiety, 5-(and-6)-carboxytetramethylrhodamine succinimidyl ester. The latter modification of fluorescent dye can be performed without significantly damaging the preformed Ag patterns. The reported technique would be useful for printed electronics. The same research group has also demonstrated photo-reactive SAM structures to create carboxyl groups upon photo irradiation [225].

Nanoarchitectonics of monolayer-type Mott field effect transistor (FET) at room temperature was demonstrated by Yamamoto and co-workers [226]. In their attempt, SAM of tetrathiafulvalene was prepared on a FET structure through anchoring on an alumina dielectric layer, where the molecule is anchored through covalent bonding of a phosphonic acid linker. The formed *p*-type monolayer FET device was then doped with 2,3,5,6-tetrafluoro-7,7,8,8-tetracyanoquinodimethane to give an ambipolar device. The observed properties such as a gate voltage shift upon doping suggested organic monolayer Mott FET-type behaviours. Temperature dependence of the doped monolayer FET exhibited an increase of activation energy with gate voltage under highly positive region, implying deformation of the Mott Hubbard band during changes of the gate voltage. It suggests the presence of Mott-insulating phase in the nanoarchitected device.

**Figure 11.**
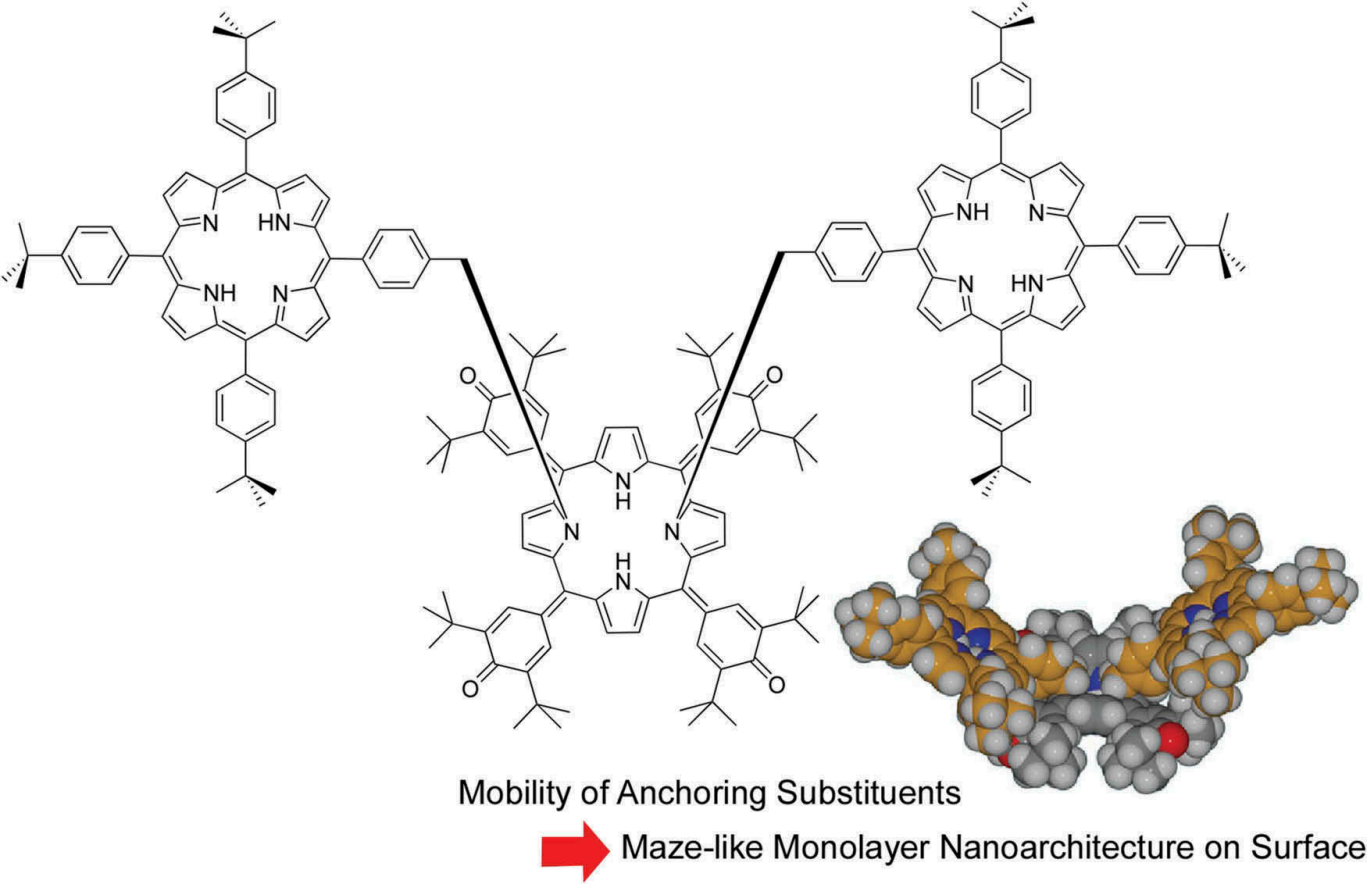
*N*-Substituted oxoporphyrinogen unit for surface-anchored rotators and unusual maze-like monolayer.

Hill and co-workers have been working on various nanoarchitectonics of oxoporphyrinogen units on the surface media [227–229]. Upon solution and sublimation self-assembly, monolayer of multi-chromophoric molecules of conjugated *N*-substituted oxoporphyrinogens was successfully prepared on the Au(111) surface (Figure 11) [230]. In that case, *N*-substituted oxoporphyrinogen unit worked as anchoring group based on strong interactive nature of *N*-substituents with the substrate Au(111). This architecture resulted in surface-anchored rotators and unusual maze-like monolayer, which would be useful for mechanical and electronic organic molecular devices.

Hill et al. also reported fabrication of binary molecular monolayers composed of tetrakis(4-pyridyl)porphyrin and tetrakis(3,5-di-*tert*-butyl-4-hydroxyphenyl)porphyrin by deposition under ultrahigh vacuum condition [231]. In one unusual case, one molecule was nicely separated within heteromolecular monolayer structures. Such molecular isolation would be useful for suppression of unnecessary molecular cross-talk in application of molecular electronic memory devices.

**Figure 12.**
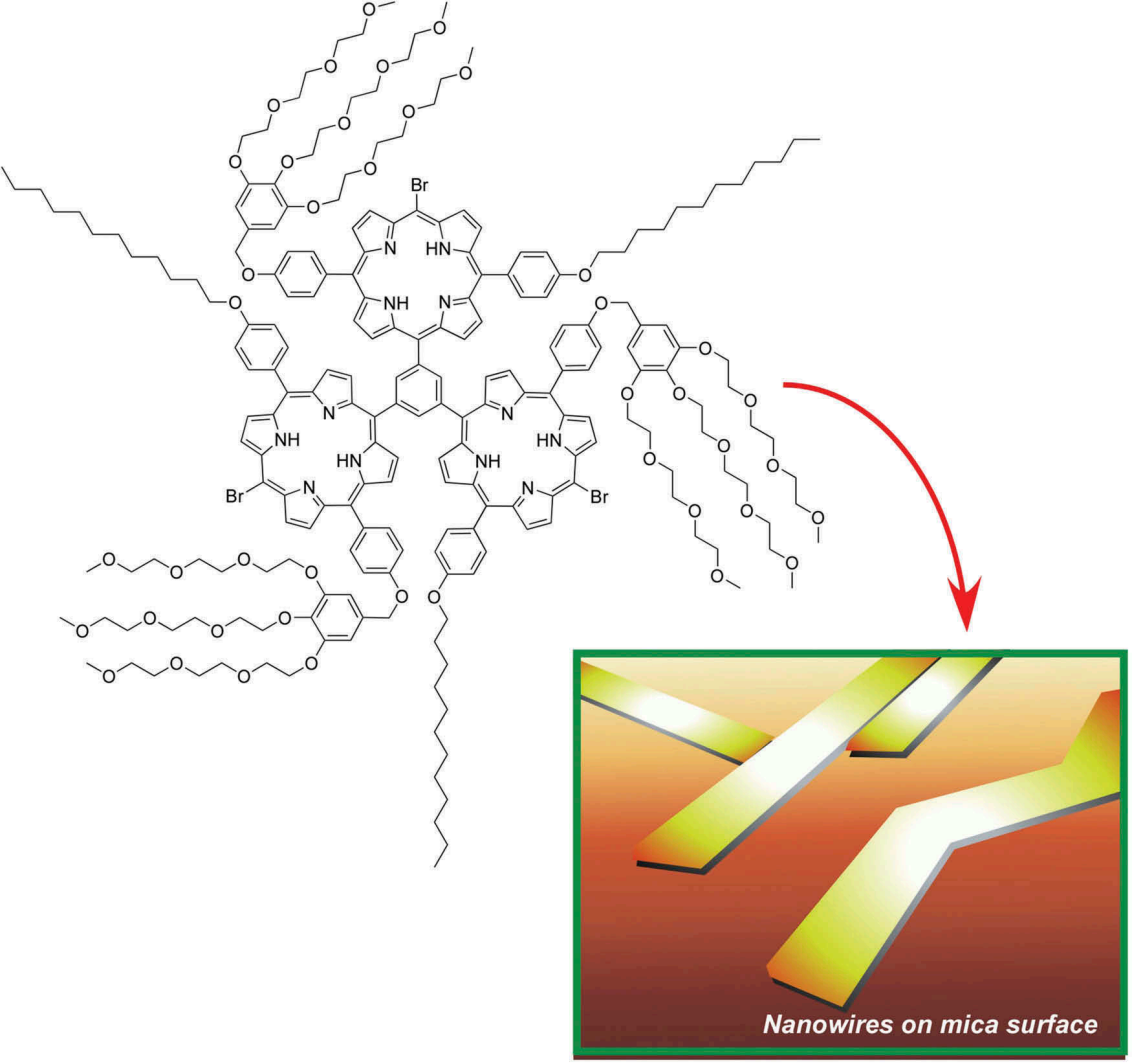
Growth of molecular nanowires of trigeminal amphiphile porphyrins on a mica surface.

Confining self-assembled structures within two-dimensional solid surface sometimes results in well-controlled growth of very thin one-dimensional nanowire assemblies. Real-time observation of molecular nanowires of trigeminal amphiphile porphyrins on a mica surface was reported by Xie, Hill, and co-workers using an atomic force microscope (AFM) (Figure 12) [232]. The trigeminal amphiphile porphyrin molecules form vesicle in solution. After the solution was deposited on mica surface, the vesicle structure disappeared concurrently with the growth of a nanowire with a height of 3.2 nm and a width of 8.5 nm. Such self-assembled nanowires could be continuously grown and manipulated, e.g. pushed, broken, and regrown. Sequential removal/regrowth resulted in the formation of new nanowires from the existing ones. The observed processes are regarded as methodology for free fusion of bottom-up self-assembly and top-down manipulation, which could be utilized for writing surface-anchored nanoscale organic circuits for molecular electronic devices.

Hill, Powell, and co-workers reported observation of self-assemblies of Fe(III) mononuclear complex with *N*-[(2-hydroxy-3-methyl-5-dodec-ylphenyl)methyl]-*N*-(carboxymethyl)-glycine disodium salt ligand on a highly oriented pyrolytic graphite [233]. Current imaging tunnelling spectroscopy confirmed a strong tunnelling current contrast at the positions of the metal ions, indicating dominant roles of metal-ion orbitals in the energy levels next to the Fermi level.

### 4.2. Thin films

Not limited to films with monomolecular level, thin films on solid surfaces are also attractive media for investigation of self-assembly. In this section, recent examples of self-assembled structures exceed monolayer level such as these films and nanocomposites on solid surface are introduced. Rodriguez and co-workers reported that composites of diphenylalanine and graphene oxide self-assembled into layered micro- and nanostructures [234]. The formed composites exhibit enhanced thermal stability with significantly reduced aqueous solubility. The formed structures on solid surface would be useful for peptide-based nanotechnology applications including energy storage and photovoltaics.

Alignments of molecules and materials are crucial in certain kinds of advanced applications and can sometimes be induced by self-assembly on solid surfaces. Selmani and Schipper demonstrated a nice example of alignment relay technique, which can transfer information of alignments from liquid crystals to molecules and materials [235]. The proposed technique actually accomplished alignment and sorting of carbon nanotubes. For this process, molecule with full triptycene was first dissolved in a room-temperature nematic liquid crystal that was deposited on rubbed aligned layer of polymeric liquid crystals. Aligned bis-triptycene molecules were transferred to the surface of indium tin oxide by simple pressing. The indium tin oxide substrate with aligned molecule at its surface was immersed in aqueous suspension of surfactant-wrapped single-walled carbon nanotubes to immobilize them on the surface of the indium tin oxide substrate. The deposited single-walled carbon nanotubes were simultaneously aligned and sorted depending both on diameter and length.

Recently, spontaneous jumping-off phenomena were discovered for condensation growing on a nanostructured superhydrophobic surface, which triggered by naturally occurring coalescence events. Boreyko and co-workers systematically investigated these jumping-off phenomena through self-assembled condensation growing on variously fabricated superhydrophobic nanostructures fabricated through arrangements of nanopillars [236]. The critical jumping diameter was successfully correlated with surface topology by energy models. The obtained knowledge would be used for antidew, antifrosting, and self-cleaning surface technology.

Siegel, Destouches, and co-workers demonstrated fabrication of three-dimensional nanostructured films by ultrafast laser-induced excitation combined with non-linear feedback mechanisms [237]. Excitation of independent optical modes at different depths in the film resulted in three-dimensional self-organization thorough thermally activated growth of nanoparticles and plasmon-induced charge separation. The demonstrated strategy can be applied for fabrication of complex layered composite systems with highly uniform large-area nano-patterns. By applying this method for useful materials such as TiO_2_, photovoltaic or photocatalytic applications are anticipated as well as multiplexed optical image encoding and security.

Tiberto et al. demonstrated magnetization reversal and microstructure through self-assembling process [238]. In their method, two-dimensional dot array can be generated by self-assembly of polystyrene nanosphere on Fe_50_Pd_50_ thin films. The observed magnetization reversal phenomena can be controlled by tuning the inter-dot distance and film thickness. Geometrical parameters for dot array and thermal annealing-induced microstructure phases are important factors for magnetic properties rather than ordering itself.

Surface self-assemblies sometimes accompany surface–surface interaction for higher level nanoarchitectures. Yamauchi and co-workers demonstrated self-contraction of layer-by-layer structure from two-dimensional motif to three-dimensional architecture between graphene oxide nanosheets and two-dimensional coordination polymers [239]. The graphene oxide nanosheets worked as nucleation sites for crystal growth of Ni-based cyanide bridged coordination polymer flakes. The formed coordination-polymer-coated graphene oxide nanosheets self-assembled into ordered lamellar nanoarchitectures. The self-assembled layered materials were finalized by thermal treatment under nitrogen atmosphere, resulting in nanomaterials with excellent electrocatalytic activity and high durability for the oxygen reduction reaction.

Jiang, Hu, and co-workers prepared a nanoglue by exploiting the strong adhesion of coordination polymers to surfaces [240]. Self-assembled coordination polymer nanoplates spontaneously stack together in the drying process of slurry of the nanoplates. The stacked nanoplates act similarly to polymeric adhesives based on collective lamellar adhesion by sandwiching the coordination polymer nanoplates between two substrates. Furthermore, coordination polymer nanoplates can be made into various shapes without help of any additional adhesives.

**Figure 13.**
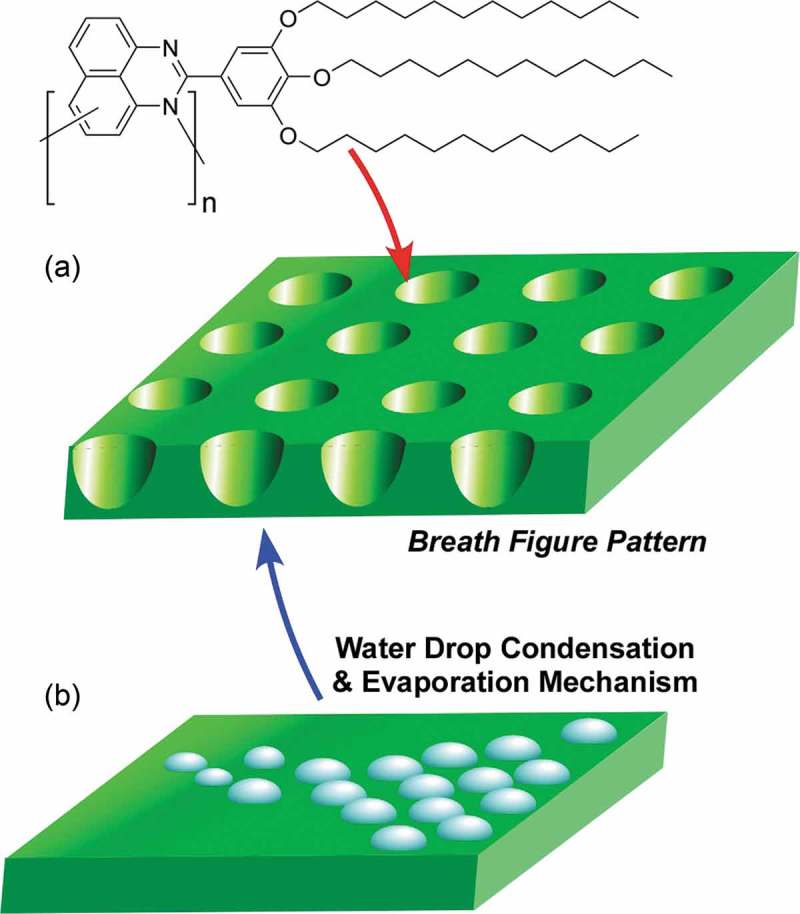
(a) Formation of breath figure motifs as hexagonally structured macroporous two-dimensional films by self-assembly of polymers from 1*H*,2*H*-dihyroperimidines derivatives. (b) Proposed mechanism for breath figure formation through water droplet condensation and evaporation.

Breath figures can spontaneously form complex structures such as honeycomb patterns. Hill et al. reported preparation of hexagonally structured macroporous two-dimensional films of polymers from 1*H*,2*H*-dihyroperimidines derivatives (Figure 13(a)) [241]. The self-assembled honeycomb structure varies uniformly with homogeneous hole sizes between 0.2 and 1 µm. They are rare examples of macroporous poly(aromatic amine). This kind of polymers has high potential for applications including sensing and electrochemical usages. Recently, Yabu has reported details of breath figure formation through water droplet condensation and evaporation in their review [242] (Figure 13(b)). Alizadeh and Nematollahi proposed the use of electrochemically assisted self-assembly technique for preparation of two-dimensional honeycomb-like mesoporous metal–organic framework thin films on an electrode surface [243]. In the formed three-dimensional hollow hexagonally packed crystals, two-dimensional honeycomb-like mesopores with the cavities are extended perpendicularly to the conducting surface. Because the electrodes modified with honeycomb pore array possess enhanced capability of electron transfer, the fabricated structures would be useful for applications such as electrochemical sensors. This method can be applied to various conductive surfaces without harmful pretreatment such as activation and chemical modification of device surface.

Surface self-assembly is powerful methodology in development of sensor devices. Goda, Miyahara, and co-workers wisely assembled plasma membranes of floating cells on the surface of ion-sensitive field-effect transistors [244]. The surface of tantalum oxide gate insulator of transistor was functionalized with oleyl-tethered phosphonic acid that can interact and immobilize plasma membranes of floating cells without affecting the cell signalling. The combined system can work as high-sensitive ion sensors on the basis of ion-accessible pores on the plasma membranes.

Kajisa and Sakata fabricated a biocompatible glucose sensor through self-assembly of a functionalized hydrogel on the surface of semiconductor-based FET [245]. The hydrogel parts were prepared by copolymerization of glucose-sensitive vinylphenylboronic acid and biocompatible 2-hydroxyethylmethacrylate as monomer components. This nanoarchitected hydrogel-based FET system can be further coupled with another device with control capability of insulin release. Three-input gate exclusive-OR logic gate devices were fabricated with hybrid bilayer of SAM structures as reported by Majima et al. [246]. This demonstrates the significance of the hybrid SAM and aluminium oxide passivation for sub-10-nm-size assembled single-electron transistors as logic devices.

### 4.3. Liquid–liquid interface

#### 4.3.1. Basic assemblies at liquid–liquid interface

Interfaces between both flexible phases such as liquid–liquid interfaces afford high motional freedom in self-assembly processes. Self-assembly nanoarchitectonics at such fully flexible interfaces sometimes creates a wide variety of self-assembled objects. In addition, liquid–liquid interfaces combine dielectric properties of both phases, resulting in self-assembly based on solubility differences.

**Figure 14.**
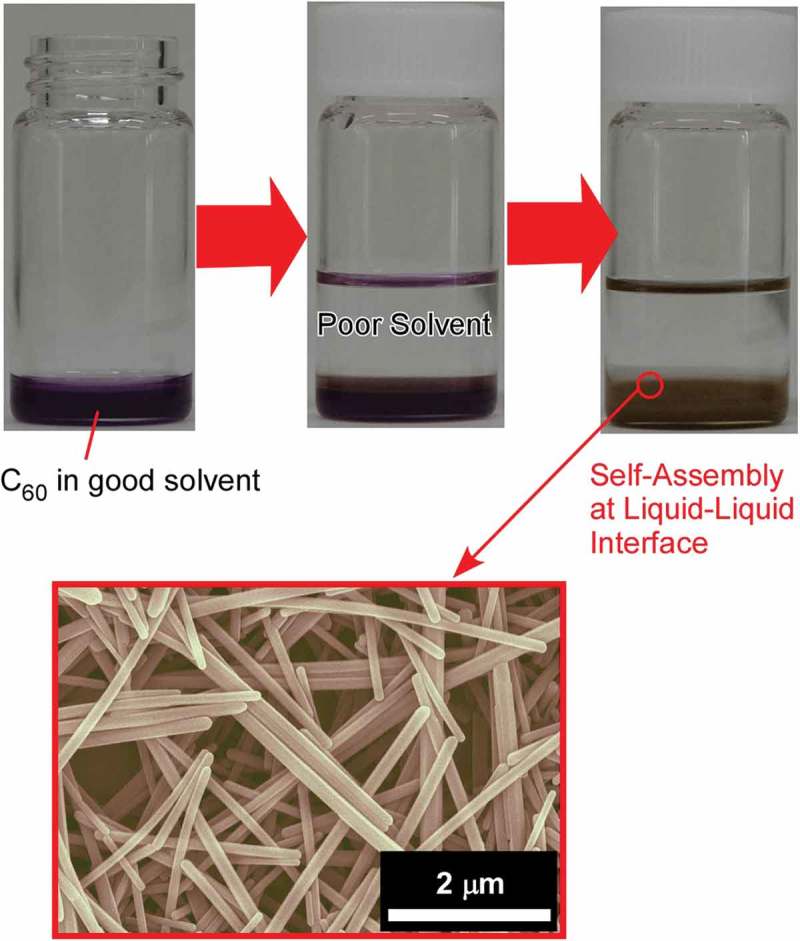
The liquid–liquid interfacial precipitation method for the production of shape-controlled fullerene crystalline self-assemblies.

The liquid–liquid interfacial precipitation method is one of the powerful methods to reflect advantages of liquid–liquid interface (Figure 14) [247,248]. As pioneered by Miyazawa and co-workers, liquid–liquid interfacial precipitation has been used for the production of shape-controlled fullerene crystalline self-assemblies. In liquid–liquid interfacial precipitation method, crystal formation is driven by the supersaturation related to very low solubility of fullerene molecules. Two types of processes, static and dynamic liquid–liquid interfacial precipitation, are available. In case of the former static liquid–liquid interfacial precipitation method, any physical disturbance is avoided to keep relatively slow process without any disturbances. On the other hand, the dynamic protocol involves application of low power sonication, agitation by hand or vertex motion, or combinations of these to the different preparation solutions. The latter process results in a homogeneous precipitation of embryo crystals of fullerenes; the crystal formation or growth is rather fast in this case and can complete within a few seconds.

In typical fullerene self-assembled crystal formation, poor solvent (usually alcohols) is slowly and carefully added into a known volume of fullerene solution (saturated solution prepared in good solvents of fullerene in most cases) so that a clear liquid–liquid interface can be formed. Since the poor solvent intermixes with good solvents, with time due to the diffusion of poor solvent towards solvent side causes unsaturation at the liquid–liquid interface. This unsaturation results in the formation of fullerene clusters (nucleus) as the direct contact of fullerene with poor solvent is energetically unfavourable. Further diffusion of alcohol towards good solvent side promotes the crystal growth by the supply of fullerene molecules from bulk to the interface, i.e. fullerene self-assembled crystal formation is diffusion-controlled. The liquid–liquid interfacial precipitation method consists of rather simple synthetic parameters such as concentration, temperature, volume ratio of solvent, and poor solvent. Tuning these synthetic conditions, shape- and size-controlled fullerene crystalline assemblies can be produced.

As nanoarchitectonics efforts for a new class of nanostructured carbon material, preparation of C_60_ crystal nanoarchitectures with bimodal pores composed both of macropores and mesopores was accomplished by Shrestha, Yamauchi, and co-workers using the liquid–liquid interfacial precipitation method at interface between isopropyl alcohol and a saturated solution of C_60_ in carbon tetrachloride–benzene mixtures [249]. The precipitation method spontaneously provided self-assembled crystals in a form of well-defined two-dimensional hexagonal nanosheet. The obtained two-dimensional crystals have bimodal pore nanoarchitectures with highly crystallized pore walls. Detailed morphologies of these two-dimensional objects can be tuned through selection of solvent combination, their ratios, and assembling temperature. The formed fullerene assemblies have enhanced electrochemically active surface areas as compared with pristine C_60_. The porosity of the material could be controlled by tuning the volume fraction of the mixed solvents carbon tetrachloride and benzene. These nanoarchitected materials would be useful for photovoltaic devices in combination with appropriate electron-donor molecules, such as porphyrin or pentacene.

Modified method of surfactant-triggered nanoarchitectonics for liquid–liquid interfacial precipitation was proposed by Shrestha et al. using nonionic surfactants, diglycerol monolaurate, and monomyristate [250]. As a basic structure, one-dimensional structure with well-defined facets was fabricated at a liquid–liquid interface between a saturated solution of C_60_ in ethylbenzene and isopropyl alcohol. This one-dimensional rod structure of C_60_ self-assembled crystals was converted into Konpeito candy-like structures with average diameter ca. 1.2 μm through addition of diglycerol monolaurate and monomyristate surfactants to the ethylbenzene solution. The prepared Konpeito-like crystals have face-centred cubic phase and their cell lattice can be tuned through selection of surfactants. Intense photoluminescence greater than pristine C_60_ and a blue-shifted photoluminescence as compared with pristine C_60_ were observed for the self-assembled Konpeito-like C_60_ crystals, implying potential usages for the optoelectronic properties. Furthermore, the Konpeito-like crystals upon heat-treatment at 2000 °C formed nanoporous carbons with a high surface area and graphitic microstructure. The heat-treated materials exhibited enhanced electrochemical properties appropriate for supercapacitance performance with long cyclic stability.

#### 4.3.2. Hierarchic assemblies at liquid–liquid interface

**Figure 15.**
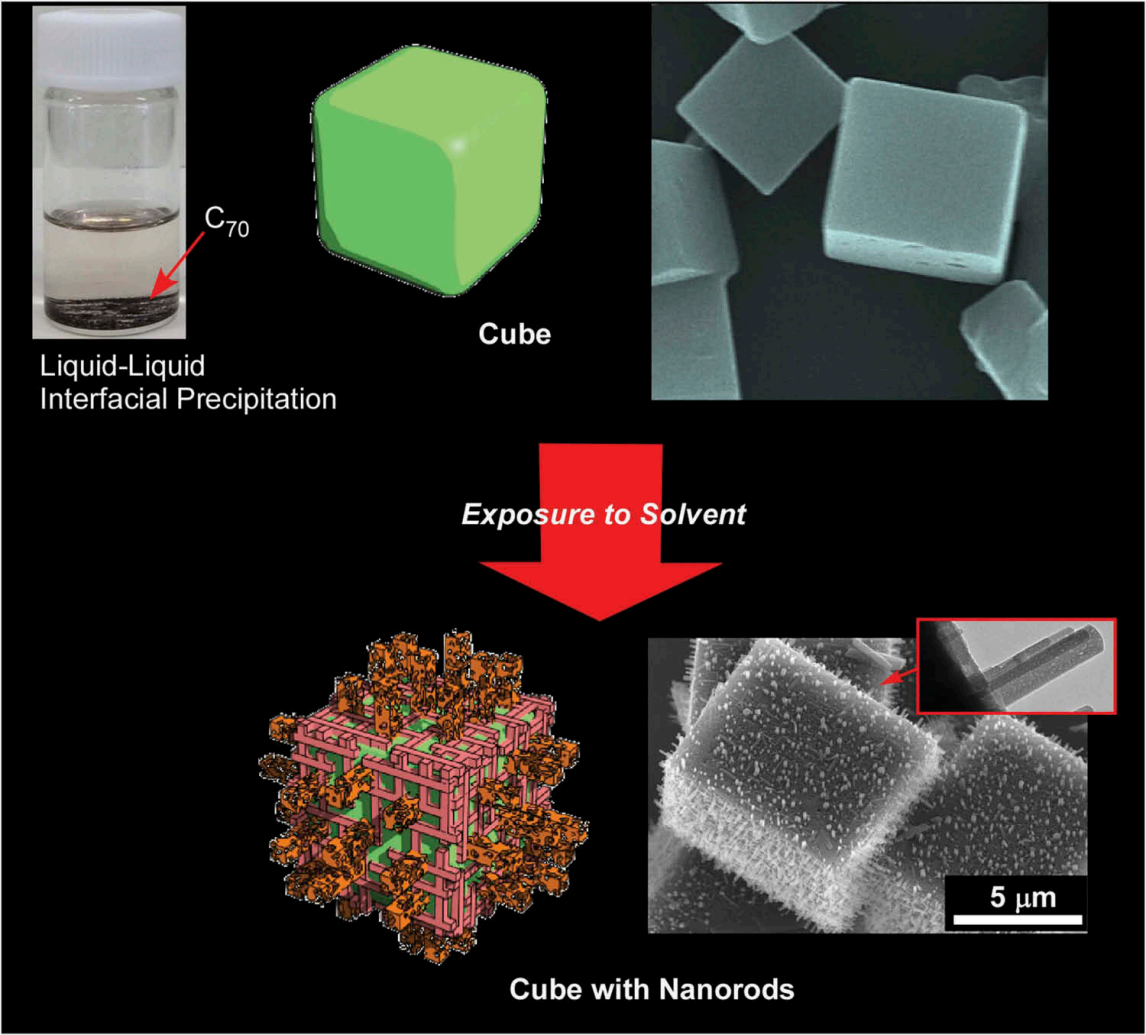
Self-assembly of C_70_ cubes from one-dimensional nanorods with crystalline pore walls fabricated through step-wise processes.

Fabrication of complicated hierarchic structures, cubic self-assembly of C_70_ with numerous one-dimensional nanorods with crystalline pore walls, through step-wise processes was demonstrated by Shrestha and co-workers (Figure 15) [251]. In the first step, cubic shape C_70_ crystals with highly crystalline structure were prepared by liquid–liquid interfacial precipitation method using C_70_ mesitylene solution and *tert*-butyl alcohol. Washing the prepared C_70_ cube with isopropanol at 25 °C as the second step induced growth of numerous C_70_ nanorods perpendicular to the faces of C_70_ cube with modulation of nanorod diameter upon washing condition selection. The formed nano-rods possess small pore structures. Excellent sensing of vapour-phase aromatic solvents was demonstrated, owing to the strong π–π interactions between guest aromatic molecules, sp^2^ carbon-rich natures of the hierarchic C_70_ assembly, and facilitated diffusion of the guest through mesoporous morphologies. In addition, an enhanced energy storage capacity larger than those observed for pristine C_70_ fullerene samples and C_70_ cubes without rods was observed probably due to an increased electrochemically active surface. Similarly, enhanced electrochemical and photoluminescence properties were observed for mesoporous C_70_ cubes prepared by modified liquid–liquid interfacial precipitation method at an interface between C_70_ in mesitylene solution and *tert*-butyl alcohol [252]. Crystalline fullerene C_70_ cubes with sharp and well-defined edges grown at 25 °C were transformed into mesoporous cubes upon gentle heat treatment at 75 °C.

**Figure 16.**
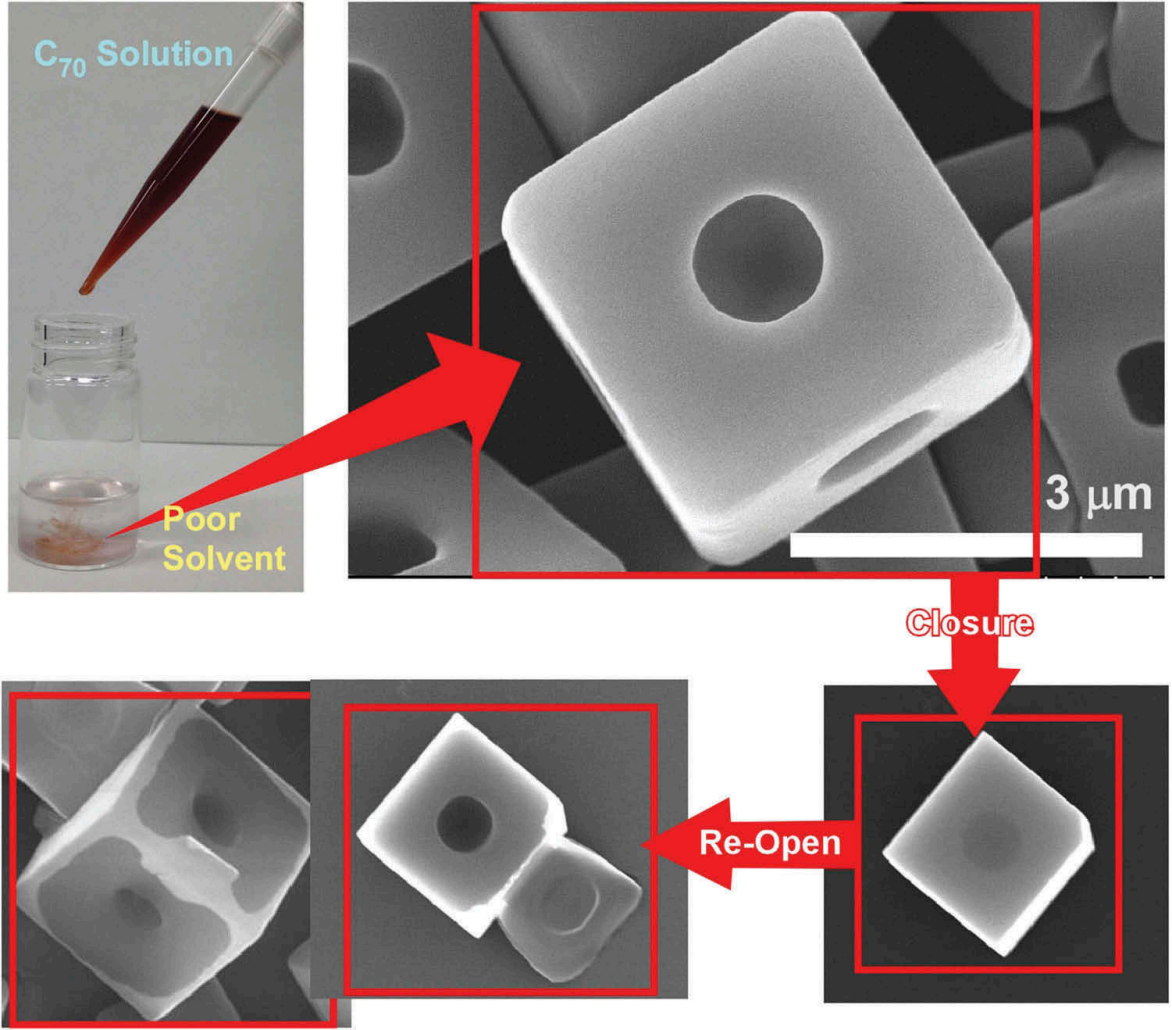
Hole-in-cube-type self-assembled C_70_ crystals with properties of intentional closing/opening actions.

Shrestha and co-workers further demonstrated hole-in-cube-type self-assembled C_70_ crystals that can have attractive properties of intentional closing/opening actions and discrimination of microscopic objects (Figure 16) [253]. The fullerene C_70_ cube with open holes, one hole at one face on the cube, was fabricated through a dynamic-type self-assembly strategy, dynamic liquid–liquid interfacial precipitation method. C_70_ molecules were first dissolved in mesitylene by applying sonication and rapidly added to poor solvent, *tert*-butyl alcohol. The hole-in-cube C_70_ assembly can be obtained as solid precipitation. Intentional closure of the open holes at the cube faces was easily accomplished just by addition of extra C_70_ molecules. The closed holes can be reopened by application of electron beam irradiation intentionally. The hole in micrometre scale at every cube face supplied environment for the accommodation of microscopic object. Interestingly, clear discrimination between resorcinol-formaldehyde polymer resin microparticle and graphitic carbon microparticle of similar size was accomplished. Graphitic carbon microparticle was preferentially trapped to the holes on the cube through strong π–π interaction. This recognition behaviour much exceed size of usual molecular recognition and would be useful for removal and detection of toxic microscopic objects such as PM 2.5 particles.

Fullerene nanorods decorated with Ag nanoparticle were synthesized by Acharya, Shrestha, and co-workers [254]. The fullerene nanorods were first prepared at an interface between methanol and a C_60_ mesitylene solution. They were covered with Ag nanoparticles by adding a solution of silver nitrate in ethanol. The self-assembled hierarchical nanohybrids were tested for usages as substrate for surface enhanced Raman scattering for the detection of target guest molecules. For example, Rhodamine 6G was detected even at nanomolar concentrations. Such self-assembled materials are expected to be used as freestanding plasmonic substrates for molecular detection in high sensitivities. Similarly, properties of surface enhanced Raman scattering for sensing were demonstrated with hierarchic cubic structures with nanorod growth prepared through self-assembly and washing processes [255].

#### 4.3.3. Bio-like assemblies at liquid–liquid interface

**Figure 17.**
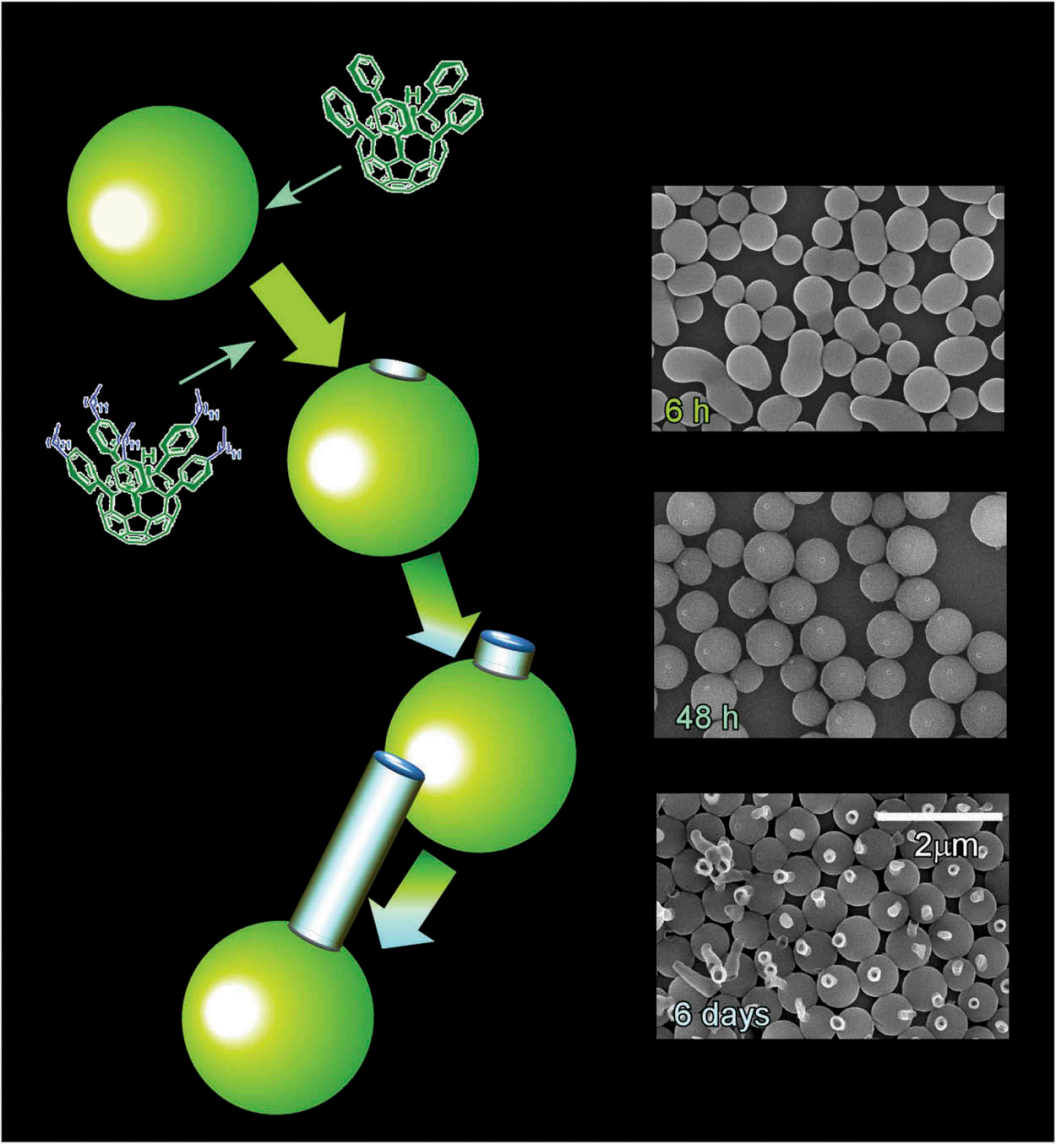
Bio-like shape-shifting self-assembly, supramolecular differentiation, through two-step process interfacial self-assembly of mixed fullerene derivatives, pentakis(phenyl)fullerene and pentakis-(4-dodecylphenyl)fullerene.

An interesting example of bio-like shape-shifting self-assembly is supramolecular differentiation, a novel concept proposed by Bairi et al. (Figure 17) [256,257]. This phenomenon was discovered through two-step process interfacial self-assembly (two-phase system of *iso*-propyl alcohol and toluene) of mixed fullerene derivatives, pentakis(phenyl)fullerene and pentakis-(4-dodecylphenyl)fullerene, followed by sonication mixing after a certain period. In this process, the egg-like spherical self-assembled objects are first formed followed by the growth of the tubular appendages after a certain period based on time-programmed supramolecular differentiation. The observed process reminds us the biological differentiation of an egg to a tadpole. The number of tails can be controlled simply by tuning the incubation time in the two-phase system. The observed supramolecular differentiation is based on controlled phase separation like embryonic development. Initially, egg-like seed particles (pentakis(phenyl)fullerene) were grown by the liquid–liquid interfacial assembly process, and phase-separated domain of pentakis-(4-dodecylphenyl)fullerene appeared only after a certain incubation time. Sonication mixing at appropriate timing induced growth of one-dimensional tails of pentakis-(4-dodecylphenyl)fullerene on the surface of pentakis(phenyl)fullerene particles. Sufficient incubation time at liquid–liquid interfacial environment resulted in creation of multiple phase-separated domains, leading to growth of multiple tails. This discovered fact based on the combination of the interfacial self-assembly and controlled phase separation can be utilized to many types of molecular systems to nanoarchitect various types of anisotropic self-assembled materials.

The use of liquid–liquid interfaces is not limited to the above-mentioned self-assembled processes. Recently, in a pioneering work, Minami et al. reported the successful regulation of myogenic differentiation at water–perfluorocarbon interfaces. In this *in vitro* culture of C2C12 myoblast cells, expression of myogenin, myogenic regulatory factors family gene, was remarkably suppressed [258]. The liquid–liquid interfaces would be powerful media for physico-chemical investigation of cellular adhesion, proliferation, and differentiation. Furthermore, transfer of cells into their Langmuir–Blodgett (LB) films was also demonstrated. This approach is beneficial for fusion research between biology and supramolecular chemistry. The obtained results on cell differentiations are totally different from those observed on hard solid surface of aligned fullerene whiskers prepared at the air–water interface. Strikingly, life at interfaces depends on the toughness of the surfaces.

### 4.4. Air–water interface (Langmuir and LB film)

#### 4.4.1. Basics of supramolecular chemistry at air–water interface

**Figure 18.**
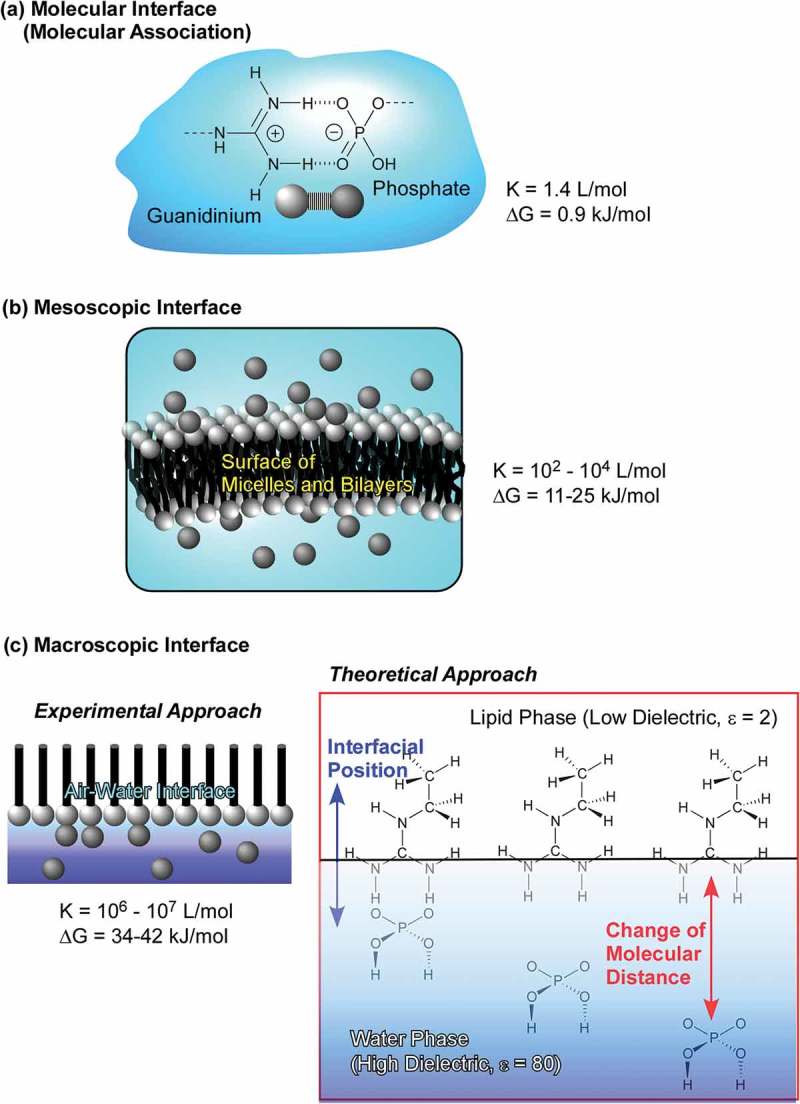
Binding constant for electrostatic hydrogen bond pair of guanidinium and phosphate (a) in water, (b) at the surface of aqueous micelles and lipid bilayers, (c) at the air–water interface.

Air–water interface is a representative of gas–liquid interface and has been extensively investigated as a medium for Langmuir monolayers and LB films. Because the air–water interface is molecularly flat medium of phases with two extremely different dielectric constants (1 for air and 80 for water under ambient condition), interactions between molecules are somehow modulated (Figure 18) [259,260]. For example, the binding constant for electrostatic hydrogen bond pair of guanidinium and phosphate in water is 1.4 M^−1^ [261] and is increased to 10^2^–10^4^ M^−1^ at the surface of aqueous micelles and lipid bilayers as disordered aqueous interface [262]. Surprisingly, the corresponding binding constants are elevated to 10^6^–10^7^ M^−1^ at the air–water interface as static and clear interface between air and water [263]. Theoretical consideration by quantum chemical approach by Sakurai and co-workers revealed that large binding constants can be regenerated by calculations through placing interacting guanidinium and phosphate pairs in the water phase significantly close to the low-dielectric-constant lipid phase [264–266]. Under these circumstances, the contribution of low dielectric constant of the lipid (air) phase favourable for the formation of electrostatic hydrogen bonding is significant even for interacting species embedded in aqueous interface.

Experimentally, enhanced molecular recognition was reported for sugars [267,268], nucleic acid bases [269,270], nucleotides [271–273], amino acids [274], peptides [275–277], and so on. These results are key to explain why biological systems accomplished hydrogen-bonding-based molecular recognition in completely unfavourable high dielectric aqueous media. Interfaces such as cell membranes, enzyme pockets, and surfaces of macromolecular objects such as DNA and polysaccharides provide appropriate media for molecular recognitions in biological systems. The air–water interface would be one of the best media for utilization of enhanced molecular interaction and molecular recognitions. Self-assembly of molecular components confined within the air–water interface is regulated by enhanced molecular recognitions, which can be also used for two-dimensional molecular patterning [278–280].

#### 4.4.2. Nanostructure formation at air–water interface

**Figure 19.**
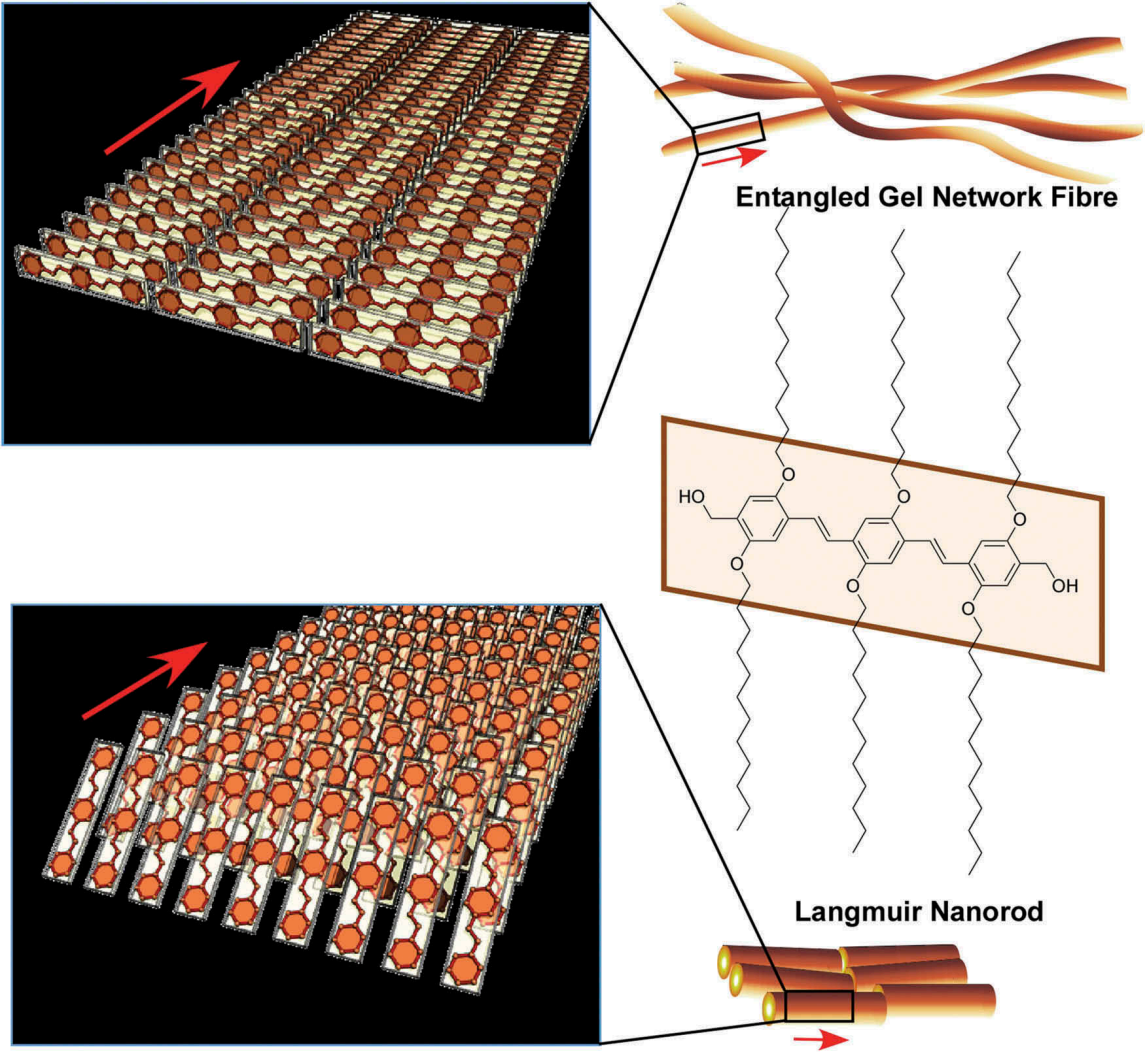
Difference of molecular orientations of *π*-gelator, oligo(*p*-phenylenevinylene) derivatives between entangled gel network fibres and one-dimensional nanorods formed at the air–water interface.

The above-mentioned characteristics of enhanced molecular interactions are available for the formation of specific nanostructures based on self-assembly at the air–water interface. Especially, molecular orientation and ordering within nanostructures can be efficiently regulated by self-assembly at the air–water interface. Sakakibara et al. successfully fabricated aligned one-dimensional nanorods of *π*-gelator, oligo(*p*-phenylenevinylene) derivatives at the air–water interface (Figure 19) [281]. The formed nanorods (length of 340 ± 120 nm and width of 34 ± 5 nm) have unusual inner orientation with oligo(*p*-phenylenevinylene) molecules arranged parallel to the length of the rods, while entangled gel network fibres formed in solutions have perpendicular arrangement of molecular orientation. The difference of molecular orientations between two-types of self-assembled materials was reflected in distinct excited-state properties upon local photoexcitation as confirmed by near-field scanning optical microscopy. Huge fluorescence enhancement was observed in the aligned nanorods self-assembled at the air–water interface. On the other hand, the entangled fibres prepared in solution led to long-range excitation energy transfer with significant fluorescence quenching. These properties are useful for the targeted applications. For example, the parallel arrangement of oligo(*p*-phenylenevinylene) molecules in the nanorods at the air–water interface would be more appropriate for charge transport, while excitation energy transfer is more suited with perpendicular arrangement of the molecules in entangled gel fibres.

Light-harvesting cellulose-based nanorods were fabricated through the self-assembly of a regioselectively pheophorbide-carrying cellulose acetate, 2,3-*O*-diacetyl-6-*O*-pheophorbide-cellulose at the air–water interface as demonstrated by Sakakibara et al. [282]. The nanorod structures were supported by a planar hydrogen bonding network. Because the assembled cellulose-based nanorods possess both optical and photocurrent generation properties, they can act as artificial light collectors similar to the photosynthetic systems. Red-shifted absorption peaks were observed for the LB monolayer film of the nanorods as compared with those observed in solution, indicating J-type excitonic coupling between the S_0_−S_1_ transition dipole moments of the chlorin Q_y_ bands. Photocurrent activities of the rods self-assembled at the air–water interface were also confirmed.

**Figure 20.**
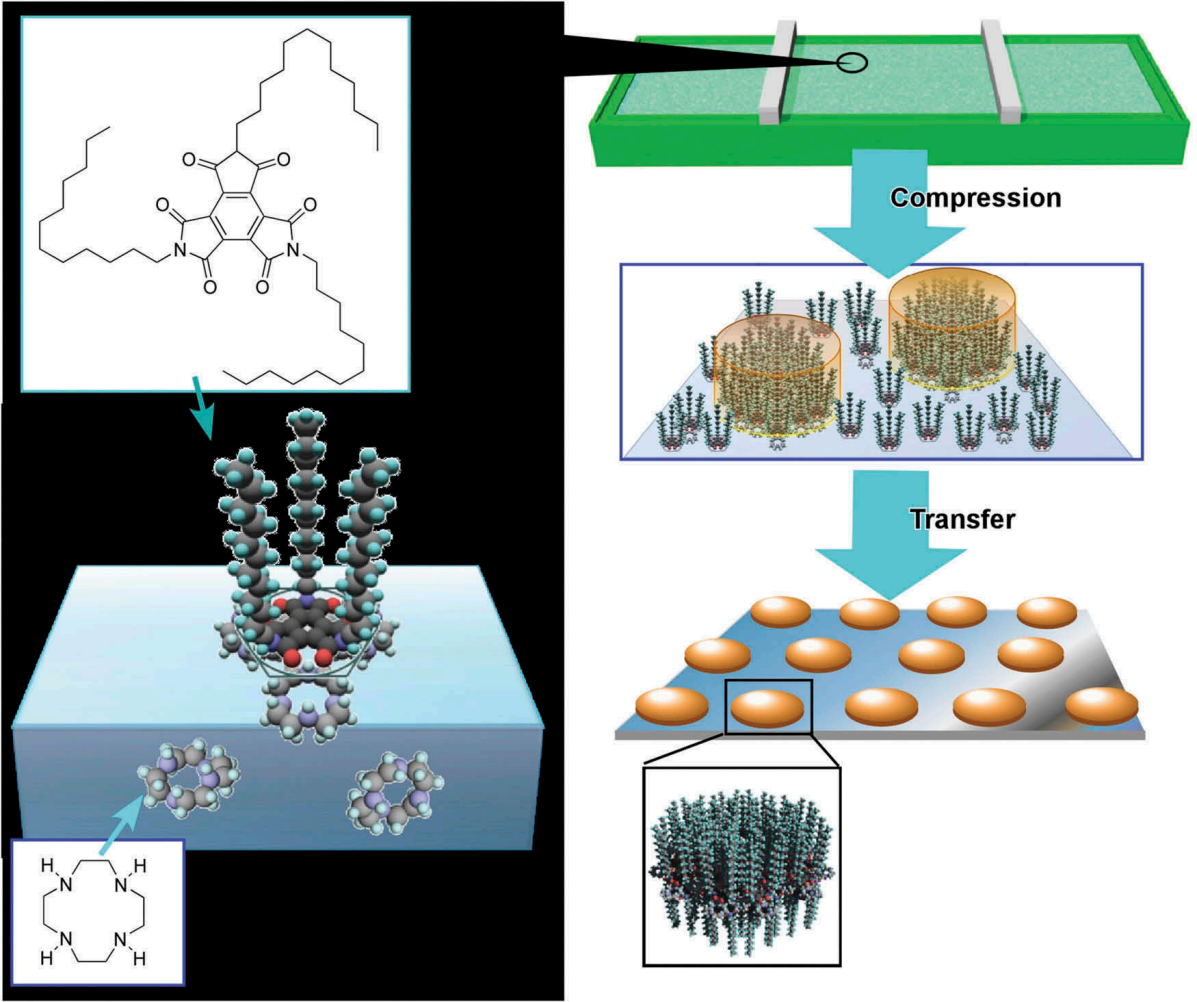
Fabrication of regularly sized nanodisks of tri-*n*-dodecylmellitic triimide with macrocyclic oligoamine, 1,4,7,10-tetraazacyclododecane though self-assembly at the air–water interface and one-touch transfer onto a solid substrate.

Mori et al. reported conventional methods to fabricate regularly sized nanodisks through self-assembly at the air–water interface and one-touch transfer onto a solid substrate (Figure 20) [283,284]. A monolayer of tri-*n*-dodecylmellitic triimide was first spread at the air–water interface, which was interacted through hydrogen bonding with macrocyclic oligoamine, 1,4,7,10-tetraazacyclododecane in subphase. Transfer of the monolayer from water surface to a mica surface through simple one-touch contact resulted in the formation of regularly sized nanodisks with a defined thickness of ca. 3 nm and tuneable diameter in the range of tens of nanometres. The formation processes to give nanosized and micro-sized regular objects are composed of a series of simple procedures including monolayer spreading, mechanical compression, and surface touching where any highly clean processes such as ultrahigh vacuum are not required.

Colloidal objects can be self-assembled at the air–water interface as seen in anisotropic self-assembly of isotropic colloidal microspheres of polystyrene within poly(*N*-isopropylacrylamide) microgels reported by Buzza, Vogel, and co-workers [285]. Corona structures by microgels were formed around polystyrene microspheres where soft repulsive force would work for controls of self-assembly processes. It can be explained by minimum energy calculations and finite temperature.

#### 4.4.3. Nanomaterial fabrication at air–water interface

**Figure 21.**
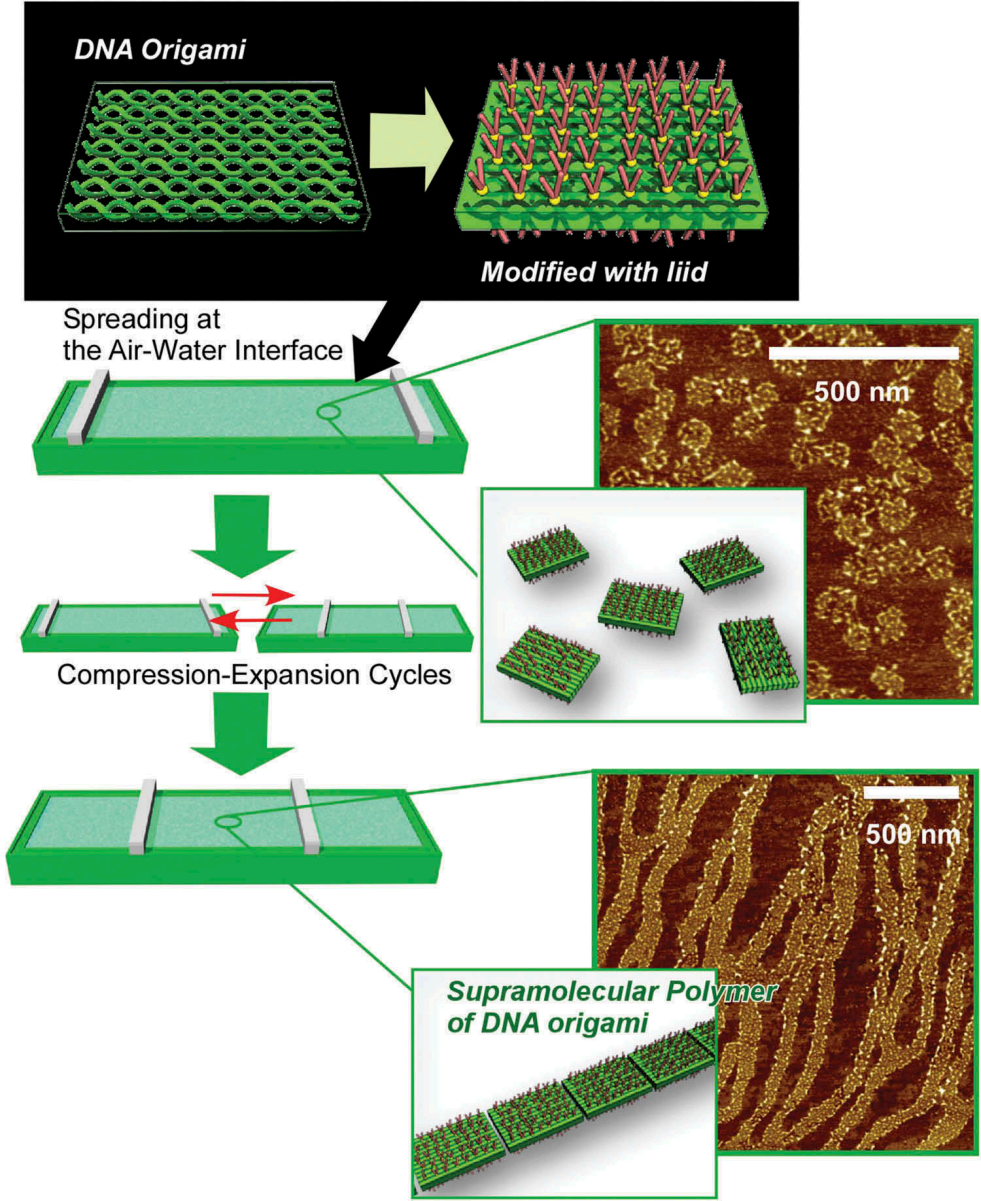
Supramolecular polymerization of the DNA origami pieces into one-dimensional upon two-dimensional mechanical stimulus motions.

The air–water interface provides opportunity to intentional alignments of biological substances as demonstrated by Yonamine et al. who successfully prepared Langmuir films of DNA wheels [286] and rectangle DNA origami [287] at the air–water interface. In the latter case, supramolecular polymerization of the DNA origami pieces into one-dimensional array was demonstrated upon two-dimensional mechanical stimulus motions (Figure 21). To prepare Langmuir monolayers at the air–water interface, negatively charged DNA origami rectangle sheets were first modified with cationic lipid, dioctadecyldimethylammonium bromide. Because the assembled complex between the DNA origami sheet and cationic lipids is soluble in organic solvents, the lipid-modified DNA origami sheets can be spread at the air–water interface. Preservation of rectangle shapes of individual DNA origami was possible even at the air–water interface. Although no changes were observed even for static standing, individual DNA origami pieces were supramolecular-polymerized into one dimensional belt upon mechanical compression and expansion cycles. The formed nanobelts keep their width identical to original sheet but were extended to a macroscopic level. The unit DNA rectangle nanosheet has unpaired DNA chains at the shorter side, and the formation of non-specific hydrogen bonds at these sides at the air–water interface induced side-specific one-dimensional supramolecular polymerization of DNA origami pieces.

**Figure 22.**
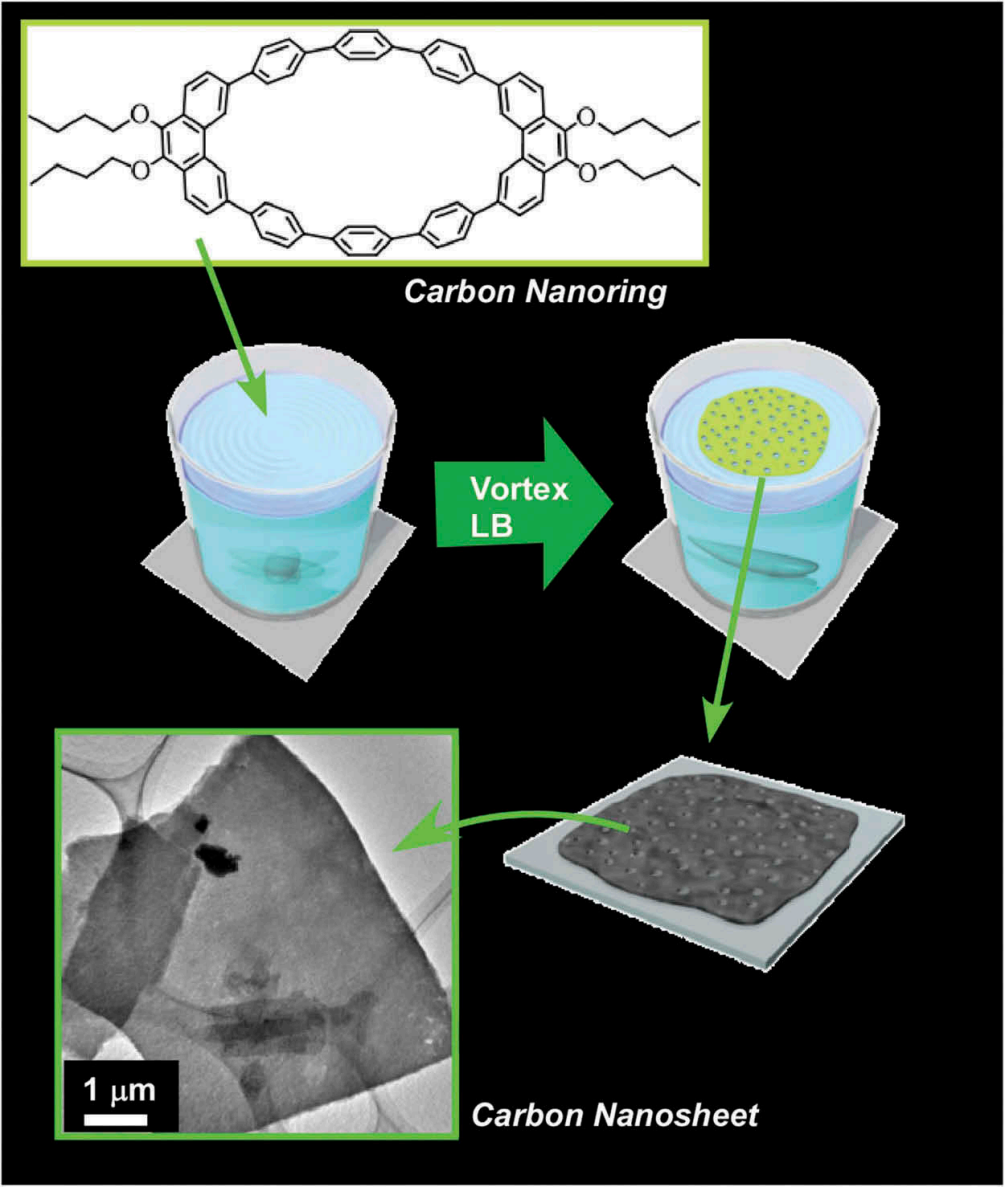
Synthesis of carbon nanosheets from anisotropic carbon nanoring by the vortex LB method.

A vortex LB method was recently invented for dynamic interfacial self-assembly. Monolayers of objects on a liquid surface can be assembled according to the rotational flow of the liquid. Aligned fullerene whiskers prepared by the vortex LB method were subjected to the controlled cell cultures [288]. Mori et al. applied this method to the synthesis of carbon nanosheets from anisotropic carbon nanorings (Figure 22) [289]. The anisotropic carbon nanoring molecules were self-assembled at the air–water interface with vortex flow to form ultrathin two-dimensional films. The assembled thin films were converted into carbon nanosheets through carbonization under inert gas atmosphere. The same processes in the co-presence of pyridine with the carbon nanoring lead to the formation of *N*-doped carbon nanosheets with unexpectedly high nitrogen contents. The fabricated *N*-doped carbon nanosheets are expected to be used in various applications including efficient catalysts for oxygen-reduction reactions for high-performance fuel cells, and high performance electrochemical supercapacitors.

### 4.5 Advanced nano/molecular system at interfaces

The final section on self-assembly at interfaces is devoted to recent developments in the molecular functions and self-assembly on surfaces. Molecular machines have been intensively studied since they became the subject of the 2016 Nobel Prize in Chemistry [290–292] and have significantly evolved. While the original studies on molecular machines were carried out in solutions, recent research also involves interfaces [293–295]. Averaged behaviours of molecular machines in solution were analysed even before the emergence of advanced molecular observation techniques such as scanning tunnelling microscopy (STM) and AFM.

#### 4.5.1. Nanocar on surface

Recent advances in molecular imaging allowed direct observation of individual components of a molecular machine and related functional molecules [296]. Because such observations have to be made for molecules at interfaces, the contribution of surface science to molecular machine research became increasingly important.

One of the attractive research targets for molecular imaging would be the investigation of motion of nanocars (or molecular cars) [297,298]. Nanocars are nanoscale objects with car-like driving motions. Observations and analyses of nanocars on a solid surface are now attractive hot research topics. In addition, the first world-wide nanocar race based on molecular image observation was held in 2017 among six teams [299–302]. As one of the attending teams, Soe, Nakanishi, and co-workers investigated their nanocar, double-paddle molecule, with driving mechanism of conformation manipulation [303]. This double-paddle nanocar is based on the structure of bisbinaphthyldurene that possesses two binaphthyl groups at both ends connected to a central durene. Three main confirmers (anti, syn, and flat) were expected for this nanocar. The syn conformer was only observed as self-assembled syn dimers in small islands just after vacuum deposition of the nanocars in a submonolayer on an Au(111) surface. A single nanocar molecule in the syn conformation can be isolated from small islands of the dimers by STM molecular manipulations, followed by conversion to the flat conformer through mechanical unfolding by STM tip. Inelastic tunnelling excitation of the double-paddle nanocar at selected STM tip apex positions results in forward movement of the nanocar on Au(111) by a step of 0.29 nm per bias voltage ramp.

#### 4.5.2. Molecular machine at air–water interface

**Figure 23.**
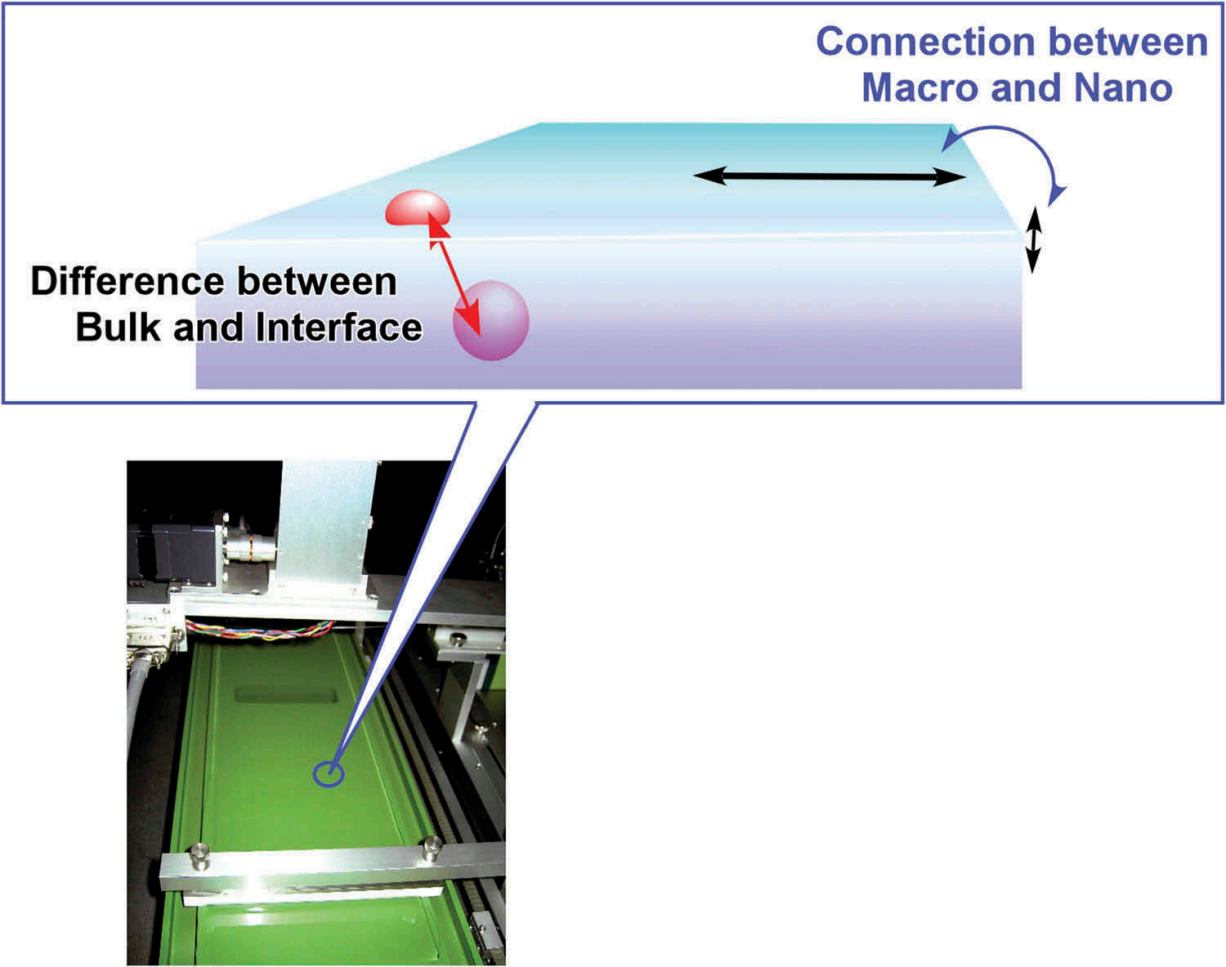
Specific aspects of dynamic interface, the air–water interface.

If molecular machines and nanocars are shifted to the air–water interface, direct observation of a single molecule becomes virtually impossible. Meanwhile, synchronized motions of laterally self-assembled molecular machines would be expected. The highly anisotropic nature of dynamic interfaces, such as the air–water interface, enables us to connect macroscopic motions with molecular functions (Figure 23) [304–307]. Lateral directions of dynamic interfaces often have macroscopic sizes, where macroscopic mechanical actions can be easily performed, including lateral compression and expansion of a two-dimensional assembly of molecular machines. On the other hand, molecules are confined at the nanometre scale along the thickness direction, and single molecular level actions are synchronized at the dynamic interfaces. Therefore, direct coupling between macroscopic mechanical motions and molecular motions is possible under these circumstances. Upon self-assembling molecular machines at a two-dimensional dynamic interface, their motions and functions can be controlled in a synchronized fashion by hand-motion-like macroscopic actions in the lateral direction of the interface; that is, at dynamic interfaces, we can operate molecular machines by our hands. This concept was named as hand-operating or hand-operated nanotechnology [308,309].

**Figure 24.**
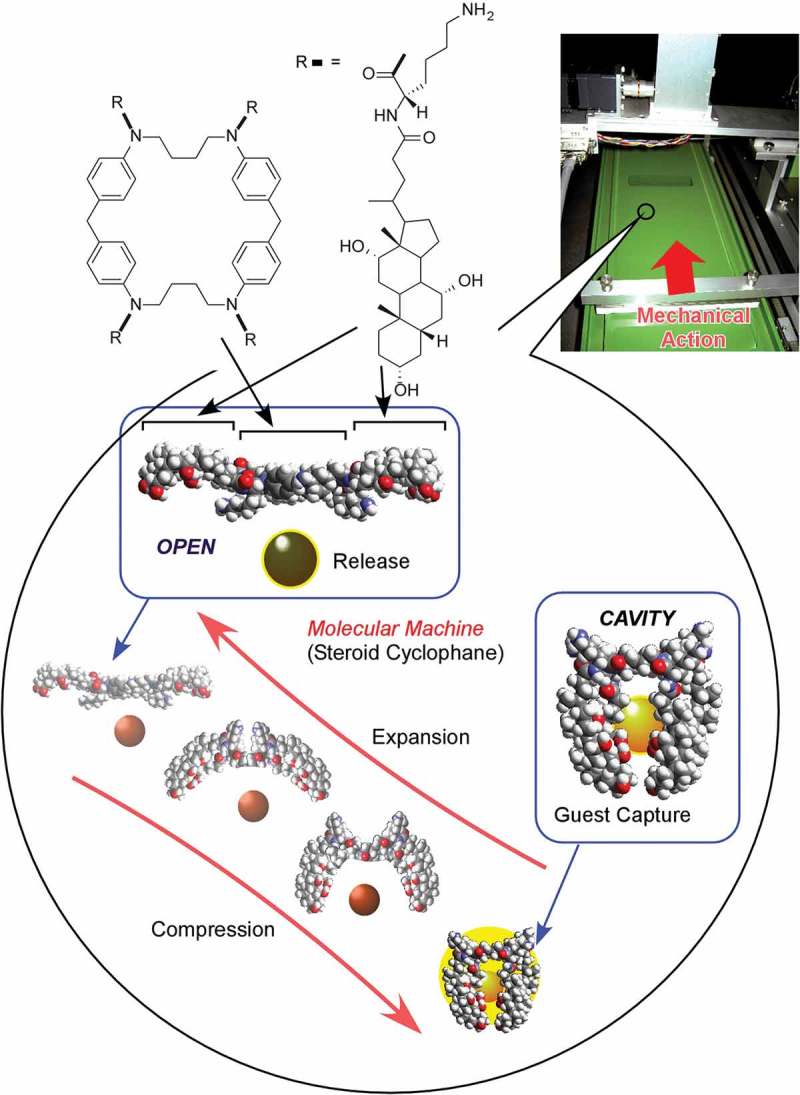
Mechanical regulation of capture and release of a target guest molecule by a molecular machine, steroid cyclophane, self-assembled at the air–water interface.

One typical example of molecular machine controlled by macroscopic mechanical motions is the mechanical regulation of capture and release of a target guest molecule by a molecular machine self-assembled at the air–water interface (Figure 24) [310,311]. The molecular machine used, steroid cyclophane molecule, has a central cyclophane ring and four rigid cholic acid walls in which the central ring and four walls are connected by flexible arms. Because the cholic acid wall possesses both the hydrophilic face and hydrophobic face, the molecular conformation of steroid cyclophane appears in its open form through favourably contacting of the cholic acid hydrophilic face at the air–water interface at low pressures. Increase of lateral pressure upon compression of the monolayer of the steroid cyclophane at the air–water interface converts the molecular conformation into its compact cavity form. This open-cavity conversion of the steroid cyclophane machines can be synchronized by macroscopic motions of monolayer expansion and compression. Capture and release of aqueous fluorescent guest molecules through cavity-open conformational conversion of steroid cyclophane were demonstrated by monitoring fluorescence emission from the captured guest molecules, revealing that guest capture and release by molecular machines can be operated by hand-like macroscopic motions.

#### 4.5.3. Molecular tuning at interfaces

**Figure 25.**
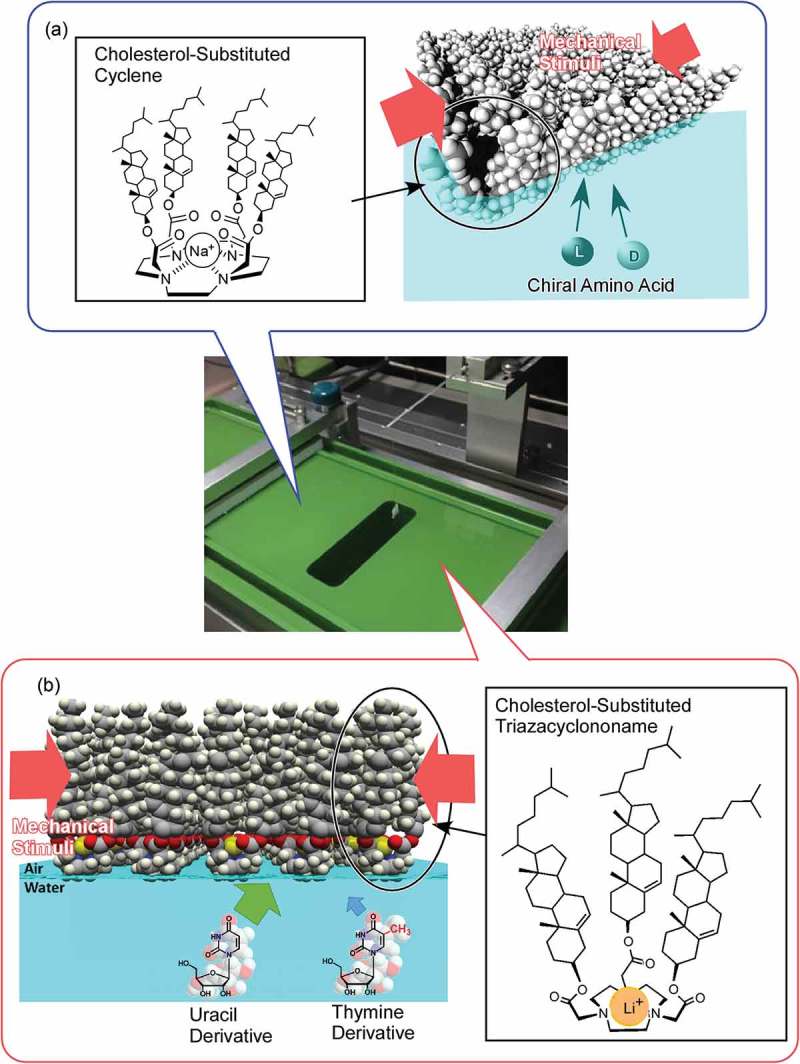
Mechanically controlled molecular recognition: (a) enantioselective recognition of amino acids by molecular receptor, octacoordinate Na^+^ complex of a cholesterol-substituted cyclen; (b) discrimination between uracil and thymine derivatives by cholesterol-substituted triazacyclononane.

The same strategy can be applied to the fine tuning of molecular receptors self-assembled at the air–water interface. Enantioselective molecular receptor, octacoordinate Na^+^ complex of a cholesterol-substituted cyclen, has the helicity originated by the chirality of the side arms, which is twisted to induce modified helicity and chirality upon gradual compression of their monolayer by mechanical lateral compression (Figure 25(a)) [312,313]. Chiral environments of the aqueous-phase-exposed surface of the cholesterol-substituted cyclen monolayer continuously shift depending on surface pressure, resulting in the pressure-regulated chiral recognition of guests in aqueous subphase. In binding of aqueous amino acid, valine, to the monolayer of the cholesterol-substituted cyclen, recognition of d-valine is preferential to l-valine at lower pressure regions. However, recognition selectivity is reversed to l-valine preferential at higher pressure regions. Therefore, we can select the desirable chiral selectivity by mechanical receptor tuning just by simple mechanical actions. Such flexible tuning of chiral selection cannot be attained by standard method of molecular recognition and its analyses such as crystallographic characterization of host–guest complex.

Efficient discrimination between uracil and thymine derivatives can be accomplished by molecular receptor tuning at the air–water interface although these resembling nucleic acid bases with only one methyl group difference cannot be usually discriminated even by naturally occurring DNA and RNA. In this case, cholesterol-substituted triazacyclononane molecules self-assembled at the air–water interface were used as molecular receptors (Figure 25(b)) [314,315]. Mechanical lateral deformation of the monolayer continuously changes the two-dimensional arrangements of hydrogen bond donor and acceptor sites, which can be further regulated through complexation of triazacyclononane to Li ions from aqueous phase. At mechanically tuned optimized condition, surface pressure of 35 mN m^−1^ and LiCl concentration of 10 mM, binding constant for uracil derivative is 64 times larger than that for the thymine derivative. Mechanical tuning of molecular receptor within their self-assembled structures at dynamic interface can compensate huge efforts in organic synthesis to covalently construct complicated molecular receptors capable of such fine molecular discrimination.

**Figure 26.**
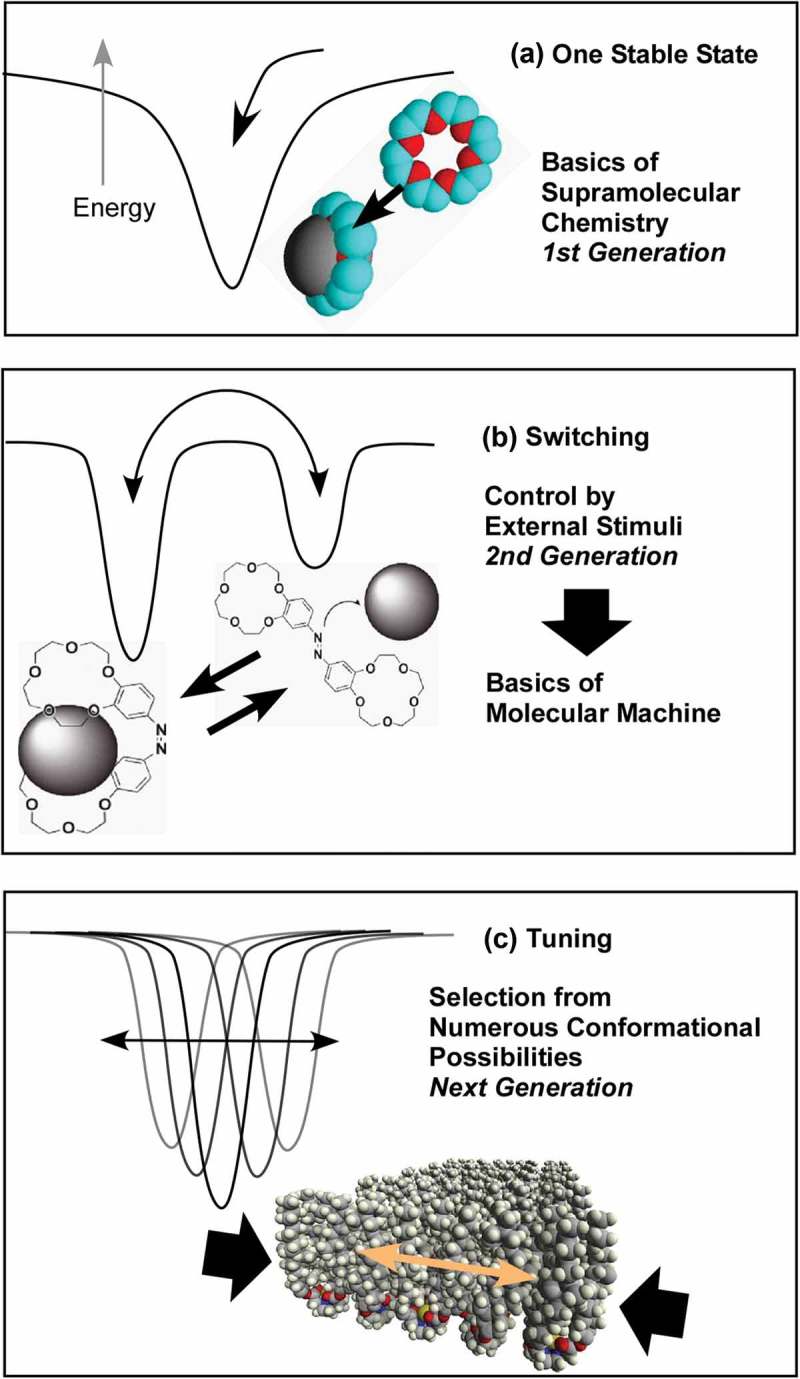
Modes for molecular recognition: (a) one stable state, (b) switching, (c) tuning.

The above-mentioned methodology for molecular recognition, molecular tuning, can be regarded as a novel category of recognition mode (Figure 26) [316–318]. As the basics for the supramolecular chemistry, which was the subject of the 1987 Nobel Prize in Chemistry, the most stable state of host–guest complex determines binding constants and binding selectivity [319–321]. This one-state mechanism is the first generation of molecular recognition mode. Important breakthrough for the molecular recognition was made by Shinkai and co-workers, who demonstrated switching of host structure upon photo-isomerization of an azo-benzene bridge of the host structure [322,323]. This work introduced the concept of switching to create two or more states for the molecular recognition and initiate molecular recognition systems controlled by external stimuli. This switching mechanism is regarded as the second generation of the molecular recognition mode. This switching of molecular systems by external stimuli can be also regarded as the basis for controlling molecular machines, the topic of the 2016 Nobel Prize in Chemistry. Unlike one-state mechanism (the first generation) and switching mechanism (the second generation), molecular tuning mechanism utilizes numerous possibilities of host structures upon continuous conformational changes. Therefore, it could be regarded as the third generation of molecular recognition. This new mode is based on the most fundamental nature of organic molecules, conformational flexibility.

Mechanical tuning of molecular receptors at dynamic interfaces can be coupled with photochemical processes between chromophores for colorimetric reporting. For this purpose, molecular receptor having a phenylboronic acid as a recognition moiety and a carboxyfluorescein as an indicator part was designed and spread as a monolayer at the air–water interface [324]. Prior to binding of guest such as glucose, the coumarin-type chromophore 4-methylesculetin was set to cap the phenylboronic acid recognition site. The efficiency of the fluorescence resonance energy transfer between carboxyfluorescein and 4-methylesculetin was regulated via fluorophore distances. Green fluorescence at around 530 nm from carboxyfluorescein fluorophore was increased through the fluorescence resonance energy transfer upon monolayer compression. However, addition of glucose into aqueous subphase replaced the 4-methylesculetin cover at phenylboronic acid by glucose, resulting in the intensity decrease of the green fluorescence. This molecular sensing based on both mechanical control and chromophore displacement is regarded as a mechanically controlled indicator displacement assay.

The natures of molecular tuning at the air–water interface were investigated with a simple model system. Because dihedral angles of binaphthyl unit can be quantitatively monitored, an amphiphilic binaphthyl molecule, molecular pliers, as a model molecule was mechanically deformed by lateral pressure at the air–water interface [325]. Changes of the torsion angle of the naphthalene planes of the molecular pliers upon mechanical compression were estimated from experimentally obtained circular dichroic activity and time-dependent density functional theory. The energies necessary for the observed structural changes were theoretically calculated and compared with the experimentally determined thermodynamic energy for the monolayer compression. Surprisingly, the energies for input and output were similar, suggesting that mechanical tuning of molecules in self-assembled states at the air–water interface is indeed energy efficient. In addition, as another nature of molecular machines at interfaces, control of digital and analogue conversion of molecular structures was accomplished by combinations of molecular machine and matrix component within two-dimensional plane [326]. Furthermore, rotational freedom depending on the organization of two-dimensional films was tested using molecular rotor molecules at the air–water interface [327].

#### 4.5.4. Advanced devices

**Figure 27.**
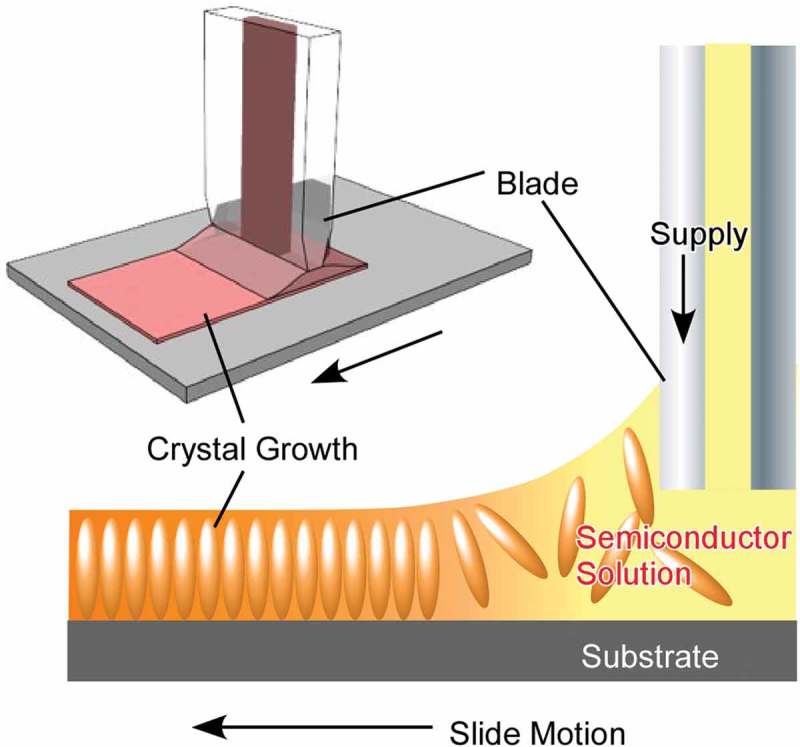
Fabrication of layer-controlled two-dimensional single crystals of organic semiconductor.

In contrast to the above-mentioned highly dynamic functions of molecular machines and molecular receptors at interfacial environments, highly organized static and regular molecular assemblies at interfacial media are useful for another advanced functional system such as device fabrications [328–332]. Owing to their versatile and tunable electronic properties, two-dimensional layered semiconductors are expected to find numerous device applications. Scale-up of the areal coverage on the surface by perfectly organized organic semiconductor molecules as homogeneous two-dimensional single crystals is crucial technique for the corresponding demands. Recently, Okamoto, Takeya, and co-workers successfully demonstrated fabrication of wafer-scale, layer-controlled two-dimensional single crystals of organic semiconductor, and their use for high-speed circuit operation. In their approach, an organic semiconductor bimolecular layer with an excellent mobility was self-assembled by a simple one-shot solution process, which possesses advantageous aspects including low contact resistance for high-speed transistor operation (Figure 27) [333]. Meniscus-driven solution method, continuous edge casting, enables organic semiconductor molecules such as 3,11-dioctyldinaphtho[2,3-d:2′,3′-d′]benzo[1,2-b:4,5-b′]dithiophene to self-assemble into a large-scale two-dimensional single crystal. Because the proposed method is available for direct deposition of organic single-crystalline two-dimensional films onto various device substrates, it would have high impact on the fabrication of large-scale integrated circuits of thin-film-based devices.

## 5. Future perspectives

This review article summarizes recent research examples of nanoarchitectonics of functional materials and systems. The earlier parts of this review illustrate self-assembling systems involving various components including small molecules, polymers, inorganic materials, and biomaterials. The almost unlimited variety of these self-assembled systems confirms the great versatility of self-assembling processes for the construction of functional nanoarchitectures. Then we provided examples of self-assembly research conducted using interfacial media, including self-SAMs, thin films on solid surfaces, assemblies at liquid–liquid interfaces, and Langmuir and LB films at the air–water interfaces. Specific characteristics and improvements in functionality were obtained depending on identity of the interface. Self-assembled materials share some basic characteristics. In contrast with the widely known three-dimensional media such as highly divided solution states, molecular motility and orientation in interfacial environments are restricted to two-dimensions often resulting in unusual organizations of self-assembled objects. Interfaces also provide interaction points for materials in different phases. Molecular interactions can be significantly modulated by the heterogeneous dielectric nature of some interfacial media. In cases of interfacial self-assembled systems, lateral macroscopic action can be coupled with molecular functionalities in a medium of molecular-level thickness. Molecular organizations and materials nanoarchitected using self-assembly at interfacial media, including aqueous surfaces and electrodes, are anticipated to be applied in advanced functional systems such as molecular machine controls, sensors, and devices. Therefore, the increasing contributions of interfacial environments and their structural characteristics will have a significant influence on the future research direction of self-assembly-coupled nanoarchitectonics.

Aspects of self-assembly-based nanoarchitectonics can be found in biomolecular evolutionary processes. It is obvious that interfacial environments such as cell surfaces, protein interior surfaces, and bio-macromolecular interfaces play critical roles in the functional expression of self-assembled bio-systems. Therefore, an idea of what might be achieved utilizing self-assembly nanoarchitectonics in artificial systems can be found in current biological systems. Several characteristics of highly evolved biological systems have not yet been fully accomplished using self-assembled nanoarchitectonics; for example, establishing highly sophisticated functional relays in self-assembled systems. As can be found in photosynthetic systems and signal transduction systems, highly efficient and directionally specified relays for function, signal, information, energy, and electron transfers operate as conventional actions in biological systems on the basis of highly evolved molecular dispositions in biological self-assemblies. Such high levels of organization have not been fully realized in artificial self-assembly nanoarchitectonics. Nanoarchitecting such high-level functional organizations is one of the future goals. Another potentially useful feature of biological systems is the selection and molecular ‘decision-making’ based on harmonization between multiple sometimes ambiguous responses. Beyond the precise relay of information, summation of ambiguity can result in circumstance-considered selection of outputs. This feature has not been accomplished in artificial self-assembled systems and it is a significant target to be accomplished using self-assembly nanoarchitectonics in the near future.

**Figure 28.**
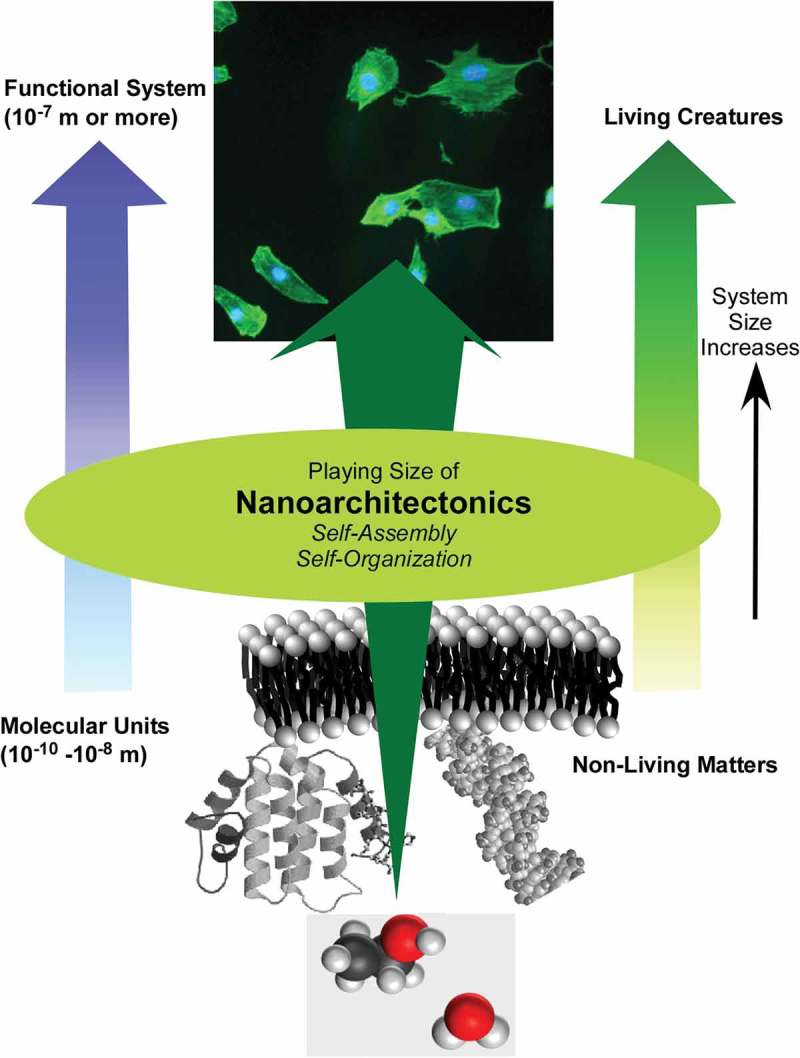
Size scale of nanoarchitectonics is similar to size range where biomolecules as non-living chemical components are assembled into systems with living functionalities.

Biological systems and nanoarchitectonics, in turn, have crucial common features (Figure 28). On a scale range from tens of nanometres to sub-micrometres, component units can be self-assembled into functional materials systems using nanoarchitectonics [334]. Similarly, biomolecules as chemical components are assembled into systems with living functionalities on a similar scale. Thus, it could be said that life is based on the assembly of molecules and construction of functional materials from nanometric units on a similar scale to that as nanoarchitectonics. This in turn suggests that the development of functional materials by self-assembly nanoarchitectonics is analogous with the evolution of living creatures from component molecules. However, while living systems took billions of years to evolve, nanoarchitectonics could be used to accomplish many of its anticipated goals within the next few decades. The important contributions of the nanoarchitectonics concept will be increasingly highlighted in future research on the science and technology of advanced materials.

Nanoarchitectonics based on self-assembly relies both on exploration of chemical designs [335,336] and self-assembly processes [337,338]. However, without creation of totally new molecules, the self-assembly processes themselves can open a huge variation of materials possibilities. For example, new materials with interesting properties can be prepared by mixing two simple amphiphilic liquids [339]. This is a very cheap, easy, fast, and new way to obtain local intermolecular self-assembly systems with enhanced proton conductivity [340], one-dimensional anomalous proton diffusion [341], peculiar solubilizing properties towards inorganic salts [342], non-Arrhenian behaviour of conductivity [343]. Even ionic liquids [344] and smart materials readily responding to a magnetic field [345] can be prepared. The similar strategy can be applied to bio-related substances such as amphiphiles, lipids, peptides, proteins, and nucleic acids, which can be obtained from natural resources without further synthesis. Therefore, the final goal described in the previous paragraph can be accomplished by sophisticated collaborations between biology and chemistry through self-assembly-based nanoarchitectonics. Chemical modification can bring molecular engineering strategies to biology [346–349], and this process can be conceptually related with the rapid evolutional progress of spatio-temporal nanoarchitectonics for science and technology of advanced materials.
